# Supplementary material for: Cohort profile: The ‘Children’s Health in Care in Scotland’ (CHiCS) study—a longitudinal dataset to compare health outcomes for care experienced children and general population children
Source: BMJ Open. 2021 Sep 14;11(9):e054664. doi: 10.1136/bmjopen-2021-054664 (PMC8442099; doi:10.1136/bmjopen-2021-054664)
Supplement: Supplementary data [file bmjopen-2021-054664supp001.pdf]

Supplement

Table S.1: Health outcomes of interest

Data set	Prior health	Outcome measures	Total events 2009-2016 *N* Valid	% Valid*
Death Records	NA	Number of deaths		
		Cause of death	824	100
PIS - Prescribing Information System	No	Number of prescriptions		
		BNF chapter/section	17,533,253	99.3
SMR00 - Outpatient Attendance	Yes	Number of admissions		
		Speciality	2,487,792	100
SMR01 - General/ Acute Inpatient and Day Case	Yes	Number of admissions		
		Condition diagnosed	412,616	100
SMR02 - Maternity Inpatient and Day Case	Yes	Number of admissions		
		Condition diagnosed	44,447	100
		Admission reason		100
SMR04 - Mental Health Inpatient and Day Case	Yes	Number of admissions		
		Condition diagnosed	8,774	99.9
		Admission reason		83.8
A&E - Accidents and Emergencies	Yes	Number of attendances per year	1,523,815	NA

*Percent with valid code for BNF chapter/section, diagnosed condition or admission reason from total recorded events. Not applicable for A&E attendances.

Allik M, *et al. BMJ Open* 2021; 11:e054664. doi: 10.1136/bmjopen-2021-054664

Table S.2: Urban-Rural Classification and Health Board of residence of CEC and CGP cohorts

	**All children with birth records** *Area of residence at birth*				**All children with birth records** *Area of residence in 2009*				**All children** *Area of residence in 2009*			
	**CGP**		**CEC**		**CGP**		**CEC**		**CGP**		**CEC**	
	**N**	**%**	**N**	**%**	**N**	**%**	**N**	**%**	**N**	**%**	**N**	**%**
**Number (%) of children**	**571,702**	**88.0**	**12,250**	**88.6**	**571,702**	**88.0**	**12,250**	**88.6**	**649,771**	**100.0**	**13,831**	**100.0**
**Urban-Rural classification**												
Large urban areas	220,713	38.6	6,164	50.3	199,694	34.9	4,853	39.6	226,405	34.8	5,349	38.7
Other urban areas	172,673	30.2	3,580	29.2	188,845	33.0	3,509	28.6	209,340	32.2	3,893	28.1
Accessible small towns	58,649	10.3	1,012	8.3	52,500	9.2	861	7.0	59,155	9.1	944	6.8
Remote small towns	15,772	2.8	363	3.0	22,037	3.9	460	3.8	25,424	3.9	505	3.7
Accessible rural	74,024	12.9	823	6.7	72,390	12.7	927	7.6	83,801	12.9	1,068	7.7
Remote rural	29,013	5.1	291	2.4	36,011	6.3	457	3.7	45,008	6.9	562	4.1
Missing	*858*	*0.2*	*17*	*0.1*	*225*	0.0	*1,183*	9.7	*638*	0.1	*1,510*	10.9
**Health Boards***												
Ayrshire & Clyde	46,869	8.2	1,356	11.1	-	-	-	-	-	-	-	-
Ayrshire & Arran	41,008	7.2	952	7.8	43,278	7.6	958	7.8	47,621	7.3	1,072	7.8
Borders	10,681	1.9	131	1.1	12,031	2.1	144	1.2	14,663	2.3	165	1.2
Dumfries & Galloway	15,581	2.7	261	2.1	16,037	2.8	263	2.1	19,064	2.9	304	2.2
Fife	39,279	6.9	640	5.2	41,013	7.2	712	5.8	45,907	7.1	796	5.8
Forth Valley	31,866	5.6	716	5.8	33,848	5.9	676	5.5	37,965	5.8	753	5.4
Grampian	54,813	9.6	1,157	9.4	55,016	9.6	1,013	8.3	65,622	10.1	1,128	8.2
Greater Glasgow (&Clyde)	103,106	18.0	2,889	23.6	132,673	23.2	3,242	26.5	148,650	22.9	3,577	25.9
Highland	24,089	4.2	390	3.2	34,323	6.0	552	4.5	41,643	6.4	636	4.6
Lanarkshire	70,439	12.3	935	7.6	73,274	12.8	1,041	8.5	89,242	13.7	1,142	8.3
Lothian	83,929	14.7	1,833	15.0	79,506	13.9	1,595	13.0	90,757	14.0	1,755	12.7
Orkney	2,145	0.4	21	0.2	2,206	0.4	23	0.2	2,709	0.4	29	0.2
Shetland	2,789	0.5	27	0.2	2,754	0.5	24	0.2	3,283	0.5	27	0.2
Tayside	41,514	7.3	893	7.3	42,602	7.5	787	6.4	48,465	7.5	891	6.4
Western Isles	2,736	0.5	32	0.3	2,916	0.5	37	0.3	3,557	0.5	46	0.3
Missing	*858*	*0.2*	*17*	*0.1*	*225*	*0.0*	*1,183*	*9.7*	*638*	0.1	*1,510*	10.9

*Health Board at birth based on 1995 Health Boards. Health Board in 2009 based on 2006 Health Boards. In 2006, NHS Argyll and Clyde responsibilities were shared between NHS Highland and NHS Greater Glasgow.

Allik M, *et al. BMJ Open* 2021; 11:e054664. doi: 10.1136/bmjopen-2021-054664

Table S.3: Residential stability of datazone between birth and 2009

	CGP with birth records		CEC with birth records	
	**N**	**%**	**N**	**%**
	**571,702**		**12,250**	
Same data zone at birth and in 2009	215,308	37.7	1,523	12.4
Moved data zone between birth and 2009	355,324	62.2	9,527	77.8
*Missing**	*1,070*	*0.2*	*1,200*	*9.8*

*Of data zones missing in the CGP cohort: 858 are missing at birth, 225 are missing in 2009 and 13 are missing in both periods. Of data zones missing in the CEC cohort: 17 are missing at birth and 1183 are missing in 2009.

Table S.4: Comparison of CEC cohort to national rates of children looked after by sex and age

	CEC cohort: Children on 2009/10 CLAS return and 2009 PC		National rates of Children Looked After*				CEC cohort as % from national rates of Children Looked After
	N	%	A) Looked after at 31 March 2009	B) Starting to be looked after 01/08/2009 - 31/07/2010	A + B	%	
Female	6,274	45.4	6,912	2,288	9,200	45.2	68.2
Male	7,557	54.6	8,375	2,571	10,946	54.8	69.0
Age (at 31st July 2009)							
0-4	336	2.4	3,157	1,796	4,953	20.7	6.8
5 to 11	6,670	48.2	5,510	1,463	6,973	36	95.7
12 to 15	5,845	42.3	5,017	1,555	6,572	32.8	88.9
16 & 17	956	6.9	1,457	45	1,502	9.5	63.6
18+	24	0.2	146	0	146	1	16.4
Total	13,831	100	15,287	4,859	20,146	100	68.7

*Source: https://www.gov.scot/publications/statistics-publication-notice-health-care-series-children-looked-statistics-2009-10/pages/1/

Allik M, *et al. BMJ Open* 2021; 11:e054664. doi: 10.1136/bmjopen-2021-054664

Table S.5: Comparison of all children in study (CEC and CGP) to Scotland's 2011 census population, age 0-19 years

	**All children in study**		**Scotland's census 2011****		
	**N**	**%**	**N**	**%**	
**All children in study (CEC and CGP), at 31 July 2009***	**663,602**				
**All children in Scotland at 2011 census**			**1,184,879**		
					% of census population
**Female**	325,712	49.1	579,949	48.9	*56.2*
**Male**	337,890	50.9	604,930	51.1	*55.9*
**Age**					
0-4	20,957	3.2	292,821	24.7	*7.2*
5 to 11	364,271	54.9	381,453	32.2	*95.5*
12 to 15	226,091	34.1	242,057	20.4	*93.4*
16 & 17	51,964	7.8	126,266	10.7	*41.2*
18 & 19	319	0.0	142,282	12.0	*0.2*

*Includes only those children in publicly-funded schools.

**Source: Scotland's Census 2011. Table DC1117SC – Age by sex, All people

Table S.6: Coding schemas for SMR00 and SMR01

Label used in tables	Codes
**Clinic specialty SMR00**	**Specialty/Discipline Codes included***
Psychiatry	G1, G1A, G2, G21, G22, G3, G4
Dentistry	D6, D61-D63, D8, DC, DE, DD
Obstetrics	E11, F3, F31, F32
**Main condition SMR01**	**ICD-10 codes included**
Diseases of the digestive system	K00-K93
Injury, poisoning and certain other consequences of external causes	S00-T98
Other injury	S10-S59, S70-T35, T51-T98
Head injury	S00-S09
Drug poisoning	T36-T50
Hand injury	S60-S69
Symptoms, signs and abnormal clinical and laboratory findings	R00-R99
Diseases of the respiratory system	J00-J99
Asthma	=J45-J46
Diseases of the genitourinary system	N00-N99
Diseases of the musculoskeletal system and connective tissue	M00-M99
Neoplasms	C00-D48
Examination, observation, etc	Z00-Z99
Obstetric	O00-O99
Endocrine, nutritional and metabolic diseases	E00-E90
Mental and behavioural disorders	F00-F99

*Clinic specialty codes are described in the SMR00 crib sheet https://www.ndc.scot.nhs.uk/Data-Dictionary/SMR-Crib-Sheets/SMR00_CRIB_07012021.pdf

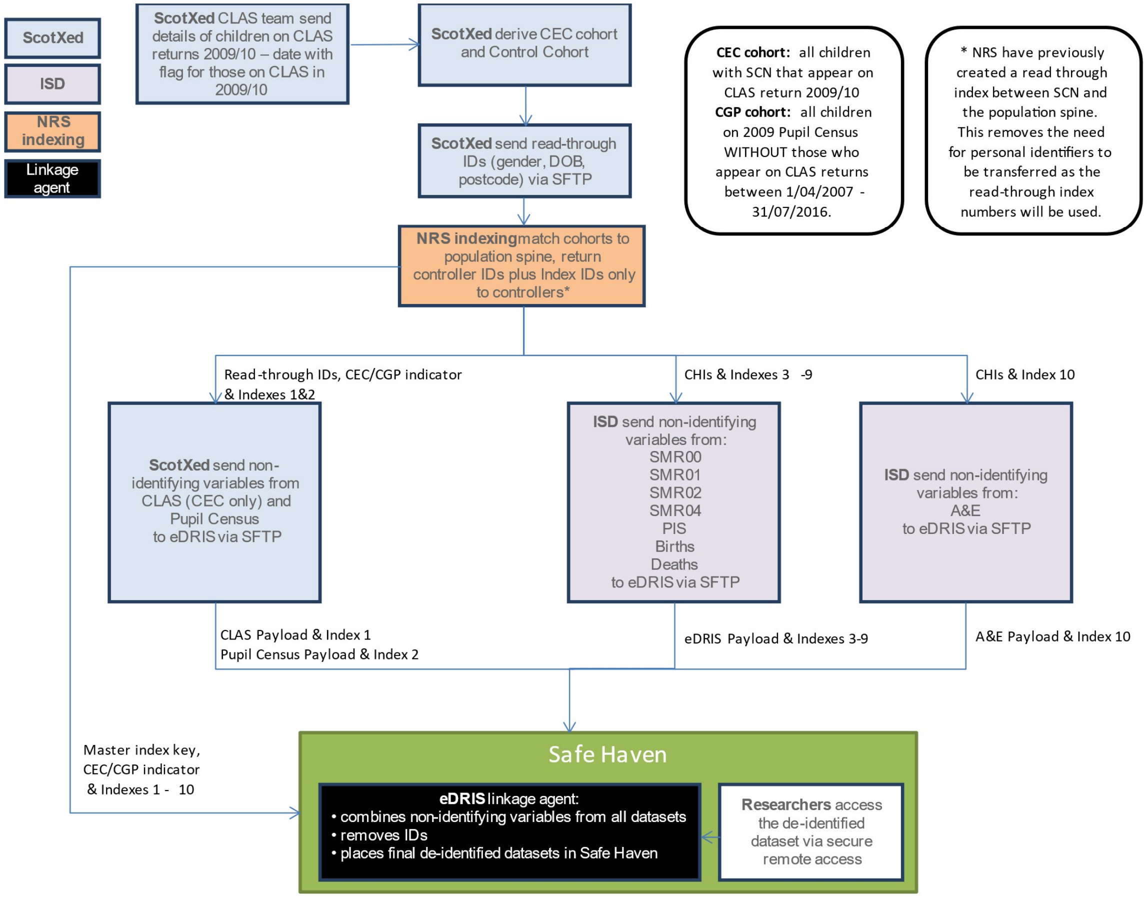

Figure S.1. Linkage diagram for the CHiCS project

CEC – care experienced children; CGP – children in the general population; SFTP – secure file transfer protocol; ScotXed – Scottish Exchange of Data; NRS – National Records of Scotland; ISD – Information Services Division (part of NHS); eDRIS – electronic Data Research and Innovation Service; SCN – Scottish Candidate Number
