# Supplementary material for: Cohort profile: The ‘Children’s Health in Care in Scotland’ (CHiCS) study—a longitudinal dataset to compare health outcomes for care experienced children and general population children
Source: BMJ Open. 2021 Sep 14;11(9):e054664. doi: 10.1136/bmjopen-2021-054664 (PMC8442099; doi:10.1136/bmjopen-2021-054664)
Supplement: Author's manuscript [file bmjopen-2021-054664.draft_revisions.pdf]

BMJ Open

BMJ Open is committed to open peer review. As part of this commitment we make the peer review history of every article we publish publicly available.

When an article is published we post the peer reviewers' comments and the authors' responses online. We also post the versions of the paper that were used during peer review. These are the versions that the peer review comments apply to.

The versions of the paper that follow are the versions that were submitted during the peer review process. They are not the versions of record or the final published versions. They should not be cited or distributed as the published version of this manuscript.

BMJ Open is an open access journal and the full, final, typeset and author-corrected version of record of the manuscript is available on our site with no access controls, subscription charges or pay-per-view fees (http://bmjopen.bmj.com).

If you have any questions on BMJ Open's open peer review process please email info.bmjopen@bmj.com

BMJ Open

# Cohort profile: The “Children’s Health in Care in Scotland” (CHiCS) Cohorts of linked Children Looked After Statistics Return, the Pupil Census, and health and mortality records to compare health outcomes for care experienced children and children in the general population in Scotland.



| | |
|---|---|
| Journal: | *BMJ Open* |
| Manuscript ID | bmjopen-2021-054664 |
| Article Type: | Cohort profile |
| Date Submitted by the Author: | 20-Jun-2021 |
| Complete List of Authors: | Allik, Mirjam; University of Glasgow, SPHSU<br>Brown, Denise ; MRC/CSO Social and Public Health Sciences Unit, University of Glasgow<br>Taylor Browne Luka, Courtney; School of Psychology<br>MacIntyre, Cecilia; Scottish Government, Data for Research Unit<br>Henderson, Marion; University of Strathclyde<br>Leyland, Alastair; MRC/CSO Social and Public Health Sciences Unit, University of Glasgow, |
| Keywords: | EPIDEMIOLOGY, Community child health < PAEDIATRICS, PUBLIC HEALTH, SOCIAL MEDICINE |
| | |



SCHOLARONE™
Manuscripts

I, the Submitting Author has the right to grant and does grant on behalf of all authors of the Work (as defined in the below author licence), an exclusive licence and/or a non-exclusive licence for contributions from authors who are: i) UK Crown employees; ii) where BMJ has agreed a CC-BY licence shall apply, and/or iii) in accordance with the terms applicable for US Federal Government officers or employees acting as part of their official duties; on a worldwide, perpetual, irrevocable, royalty-free basis to BMJ Publishing Group Ltd ("BMJ") its licensees and where the relevant Journal is co-owned by BMJ to the co-owners of the Journal, to publish the Work in this journal and any other BMJ products and to exploit all rights, as set out in our licence.

The Submitting Author accepts and understands that any supply made under these terms is made by BMJ to the Submitting Author unless you are acting as an employee on behalf of your employer or a postgraduate student of an affiliated institution which is paying any applicable article publishing charge ("APC") for Open Access articles. Where the Submitting Author wishes to make the Work available on an Open Access basis (and intends to pay the relevant APC), the terms of reuse of such Open Access shall be governed by a Creative Commons licence – details of these licences and which Creative Commons licence will apply to this Work are set out in our licence referred to above.

Other than as permitted in any relevant BMJ Author's Self Archiving Policies, I confirm this Work has not been accepted for publication elsewhere, is not being considered for publication elsewhere and does not duplicate material already published. I confirm all authors consent to publication of this Work and authorise the granting of this licence.

For peer review only

# Cohort profile: The “Children’s Health in Care in Scotland” (CHiCS) Cohorts of linked Children Looked After Statistics Return, the Pupil Census, and health and mortality records to compare health outcomes for care experienced children and children in the general population in Scotland.

For peer review only

Mirjam Allik[1*], Denise Brown[1], Courtney Taylor Browne Lūka[2], Cecilia Macintyre[3], Alastair H Leyland[1], Marion Henderson[4]

[*] Corresponding author, email: mirjam.allik@glasgow.ac.uk

[1]MRC/CSO SPHSU
University of Glasgow
Berkeley Square
99 Berkeley Street
Glasgow G3 7HR

[2]School of Psychology
University of Glasgow
62 Hillhead Street
Glasgow G12 8QB

[3]Data for Research Unit
Statistics and Data Access Division
Scottish Government
1-F South, Victoria Quay, Edinburgh, EH6 6QQ

[4]School Of Social Work and Social Policy
University of Strathclyde
Lord Hope Building
141 St James Road
Glasgow
G4 0LT

Word count: ~3300

Keywords: care experienced children, looked after children, health inequalities, administrative data, data linkage



For peer review only - http://bmjopen.bmj.com/site/about/guidelines.xhtml

## Abstract

**Purpose:** The Children's Health in Care in Scotland Cohorts were set up to provide first population-wide evidence on the health outcomes of care experienced children (CEC) compared to children in the general population (CGP). To date, there are no data on how objective health outcomes, mortality and pregnancies for care experienced children are different from children in the general population in Scotland.

**Participants:** The care experienced children cohort includes school-aged children who were on the 2009/2010 Scottish Government's Children Looked After Statistics (CLAS) return and on the 2009 Pupil Census (PC). The children in the general population cohort includes those who were on the 2009 PC and not on any of the CLAS returns between April 1st, 2007 – June 31st, 2016.

**Findings to date:** Data on variety of health outcomes, including mortality, prescriptions, hospitalisations, pregnancies, and Accident and Emergency (A&E) attendances, were obtained for the period August 1st, 2009 – June 31st, 2016 for both cohorts. Data on socioeconomic status (SES) for both cohorts were available from the Birth Registrations and a small area deprivation measure was available from the PC. CEC have, on average, lower SES at birth and live in areas of higher deprivation compared to CGP. A higher proportion of CEC have recorded events across all health data sets and they experienced higher average rates of mortality, prescriptions, and hospitalisations during the study period. The reasons for contacting health services vary between cohorts.

**Future plans:** Age-standardized rates for the two cohorts by sex and area deprivation will be calculated to provide evidence on population-wide prevalence of main causes of death, reasons for hospitalisation and types of prescription. Event history analysis will be used on matched cohorts to investigate the impact of placement histories and socioeconomic factors on health.




For peer review only - http://bmjopen.bmj.com/site/about/guidelines.xhtml

# Article Summary

## Strengths and limitations of this study

- The CHiCS project is the first population-wide longitudinal data collection in the UK that links administrative data on social care, births, deaths, hospitalisations and prescriptions to compare the health of care experienced children to children in the general population.
- The study looks at a wide range of health outcomes, including in- and outpatient hospitalisations, prescriptions, A&E attendances, pregnancies and mortality.
- Care experienced children had higher average rates of mortality, prescriptions, and hospitalisations during the study period compared to children in the general population, and the differences in health between the two cohorts are most notable for mental, sexual and reproductive health.
- The weaknesses of the study include errors in the individual data sets and in data linkage (affecting about 3% of the study population), and the exclusion of children who were not in school or were in independent schools (approximately 4% of all school-aged children).



For peer review only - http://bmjopen.bmj.com/site/about/guidelines.xhtml

## Introduction

There is very little quantitative evidence on how the health of care experienced children[1] (CEC) compares to other children in Scotland and in the UK. Invariably, the evidence that is available suggests that care experience is related to poorer health,[1–4] but often this is based on small sample sizes or without comparison to children who have not been in care.[2,3] The only population-wide evidence on the effects of care experience on health in Scotland (and the UK) comes from a linked administrative data study on dental health.[4] The study indicates considerable complexity in how different care experiences are related to health. Overall, care experienced children experience worse dental health outcomes compared to children who have not been in care, but there are significant differences in health outcomes by care type. For example, urgent and non-urgent dental needs were highest for those in home or kinship care and lower for children in foster care.

The cited research also shows that CEC are more likely to live in deprived areas compared to those who have not been in care, with half of all children in home and kinship care living in the most deprived areas. Importantly, differences in health outcomes between children persist after accounting for area deprivation.[4] Many previous studies have often not been able to account for area deprivation and family socioeconomic status (SES),[1] something that has a substantial effect on both health outcomes[5] and the chances of experiencing social care.[6]

As there is very limited evidence on which health outcomes are most likely to differ between children who have and who have not experienced care, this project includes a wide variety of health measures. In the UK, researchers have reported higher mortality,[7] poorer self-rated general and mental health,[8] and higher rates of some physical illnesses (epilepsy, cystic fibrosis, and cerebral palsy)[9] among care experienced children. Research in Sweden, Finland and Canada underline high rates of mental health related problems like suicide and suicide attempts, psychiatric disorders and substance abuse among the care experienced population.[10–14] Other international research has shown evidence of higher rates of avoidable deaths (e.g. from causes that could have been prevented by timely medical care, or homicide and unintentional injuries)[15] and emergency department visits[16] among children in foster care.

There is also evidence of higher pregnancy rates among young women in care.[17] Based on these findings and our discussions with the Centre for Excellence for Children's Care and Protection (CELCIS) and the Scottish Government, we also decided to include data on pregnancies for the young women in our cohort.

Administrative data linkage is the only feasible way of comparing a wide range of objective health outcomes between CEC and children in the general population (CGP) nationally, and in a representative way. Very few available data sets in Scotland and in the UK include details of both out of home or formal care and indicators of health. National surveys of children's health (e.g. Health Behaviour in School-aged Children study) and Scotland's Census include indicators of out of home and residential care, but these sources do not include any information on care history, or placement types and lengths. In Scotland, a child can also be "looked after" while living at home with their parent(s) under a home supervision order. For survey data, this means that it is not always possible

[1] 'Looked after children' is the statutory term for children in the care of local authorities, but often this excludes children who have left care. The term 'care experienced children' is now widely used to describe any person who has experience of being in care, regardless of their placement length, type or age.



For peer review only - http://bmjopen.bmj.com/site/about/guidelines.xhtml

to distinguish whether the child is formally considered "looked after" while living at home or with relatives. Finally, some surveys have too few CEC to analyse health outcomes for this subpopulation.

The lack of quantified evidence on the health of care experienced children has long been recognized as a major obstacle to evidence based policy making in the field.[18] The Children's Health in Care in Scotland (CHiCS) study provides the first robust nationwide evidence, with longitudinal data on care histories for CEC and a wide range of health outcomes for both cohorts of children. Improved knowledge about the health outcomes of CEC, particularly in comparison to CGP, will assist with the allocation of services by identifying precise areas of heightened needs, and will inform future research seeking to understand health inequalities between CEC and CGP.

## Cohort description

The CHiCS cohorts follow a range of health outcomes for two groups of school-aged children in publicly funded schools in Scotland; CEC and CGP (i.e. not care experienced). All school-aged children on the 2009/2010 CLAS return and all children in the 2009 Pupil Census (PC) were included in the baseline cohorts. The CEC cohort was defined as all those children who were on the 2009/2010 Scottish Government's Children Looked After (CLAS) return, collected between August 1st, 2009 and July 31st, 2010. The care histories of CEC were followed up to July 31st, 2016 longitudinally, linking seven CLAS returns. The CGP cohort was defined as children who were on the 2009 PC, taken on September 21st, that have not experienced care (not appeared on any of the CLAS returns from the first collection date on April 1st 2007 to the most recent date included in our study on July 31st 2016).

The two cohorts of children were linked to all other data sources using the Scottish Candidate Number (SCN) present on the PC. The linkage of CLAS records to health is only possible via the SCN, and CEC who were not in school in 2009/2010, or had an invalid or missing SCN could not be included in the cohort. The 2009/10 CLAS return was chosen as the baseline cohort as the completeness of SCN on the CLAS return became high enough to make linkage between the CLAS return, the PC and health data representative. Details of SCN completeness for a single CLAS return has been published previously[19] and for our study this was improved further by the longitudinal linkage of multiple CLAS returns.

The individual level linkage process of the 10 data sources is described in Supplement Figure S.1 and follows the steps outlined previously for research on dental health.[19] Data cleaning steps and the size of the two cohorts are described in Figure 1. The key data sets in deriving the cohorts were the CLAS return and the PC. Twins and triplets were removed using information from Birth Registrations (the linkage is based on postcode, date of birth and sex, thus twins living at the same address are difficult to reliably match).

In total, 663,602 school-aged children were included in the study. Of these 13,831 (2.1%) were identified as CEC and 649,771 (97.9%) as CGP. There was a higher proportion of male CEC than male CGP (54.9% compared to 50.8%; see Table 1). On average, CEC tended to be older than CGP. CEC were aged 11 years and 3 months on average (mean age in months=135, SD=42.2) and CGP aged 10 years and 10 months on average (mean age in months=130, SD=43.0), with a higher proportion of children in the CEC cohort born in the early 1990's. Differences in the age and sex distributions for all CEC and GCP with birth records is comparable to the differences in the age and sex distributions for all CEC and CGP. This suggests that children with birth records available are representative of all children in the study.



For peer review only - http://bmjopen.bmj.com/site/about/guidelines.xhtml

### Individual level socioeconomic profile

In total, 88.6% and 88.0% of CEC and GCP respectively had birth records available with information including mothers' age at the time of birth and parental employment status (see Table 1, columns on data for children with birth records). On average, mothers of CEC were younger at birth (24.5 years old compared to 28.3 years). In total 56.1% of mothers of CEC were aged under 25 years old, compared to 26.1 % of mothers of CGP.

For children born before 1996, only one parent's occupation was recorded at birth (father's occupation if married, otherwise mother's occupation) and we therefore report parental employment status for children born before 1996. Parents of CEC were less likely to be in employment than parents of CGP (71.2% in employment compared to 92.8%). From 1996 onwards, both mother's and father's occupation were recorded for all births registered by married couples or for births that were jointly registered by unmarried couples, so we report both mother's and father's employment status. Only 40% of mothers of CEC, born after 1995, were in employment compared to 78% of mothers of CGP. Fathers of CEC were also less likely to be in employment than fathers of CGP. Note also that the occupational status of CEC fathers was more likely to be missing (23.2% compared to 5.7%) indicating more absence amongst CEC fathers.

### Area level socioeconomic profile

Birth records include information on area of residence at birth (2001 data zone) and the PC records information on area of residence (2001 data zone) at the start of the study in 2009. We linked data zones at birth to the 2004 Scottish Index of Multiple Deprivation (SIMD) and linked data zones at the start of the study in 2009 to SIMD 2009v2. Table 2 gives the SIMD quintiles for area of birth for all children with birth records, for area of residence at start of study in 2009 for all children with birth records, and for area of residence at start of study in 2009 for all children in the study. In total, 59% of CEC (with birth records) lived in the most deprived quintile of deprivation compared to 25% of CGP at birth. By 2009, 41% of CEC (with birth records) were living in the most deprived quintile, perhaps reflecting residential moves into a less deprived area following birth and being taken into care.

The data zones can also be linked to Health Boards and to the Scottish Government Urban Rural Classification 2003/04 (at birth) and 2009/10 (in 2009), used to classify small areas as urban, rural or remote (Supplementary Table S.1). In terms of the urban-rural classification there was a shift for CEC away from large urban areas between birth and 2009 (50% in large urban areas at birth compared to 40% in 2009 for CEC with birth records). CEC were also more likely to have changed data zones between birth and the start of the study in 2009 (Supplementary Table S.2). Only 12.4% of CEC were living in the same data zone of residence at birth by the beginning of the study. CEC were also more likely to have missing information on data zone of residence (mostly based on data zone from the PC rather than from birth records), highlighting one of the difficulties in studying hard-to-reach populations.



For peer review only - http://bmjopen.bmj.com/site/about/guidelines.xhtml

**National comparisons**

Our cohort of CEC include those who were on the 2009/ CLAS return and in the 2009 PC. To give an estimate how well we capture the whole population of CEC and CGP in Scotland, we compare all children in our CEC cohort to Scottish Government National Children's Social Work Statistics (Supplementary Table S.3). Compared to national statistics on children in care, we see a very similar sex distribution with around 55% males and 45% females in care. Our age distribution varies in the youngest age group (0-4 years) as we include only school-aged children in our cohort and therefore only have a small proportion of four-year olds who were registered for school. For ages 5-15 we are capturing very similar numbers of CEC compared to the published national statistics.

We also compared all children in our study (CEC and CGP; n=663,602) to Scotland's 2011 census population aged 0-19 years old (Supplementary Table S.4). As a % of the 2011 census population we have captured a high proportion of children of compulsory school age (95.5% of 5–11-year-olds and 93.4% of 12-15-year-olds). Note that for ages 5-15 we are including about 95% of the population in Scotland. We also have captured 98.1% of the 2009 Pupil Census population of 676,740 pupils.[20] In conclusion, these comparisons suggest that our two cohorts are capturing a very high proportion of all school-aged children in Scotland and our study is representative of this population.

## Patient and Public Involvement Statement

We collaborated with CELCIS when planning and designing this research project. We have set up an Advisory Group including representatives from children's charities and public authorities responsible for the welfare of children and care experienced children to help guide and contextualize the research, and undertake knowledge exchange and user engagement programme. The planned knowledge exchange and user engagement programme will act as patient and public involvement and will be undertaken as the research progresses in 2021/2022.

## Findings to date

Health outcomes for the CHiCS cohorts will be examined over the 7-year period between 2009 and 2016. The health data include the number of and diagnoses relating to hospital admissions (from Scottish Morbidity Records; SMR01/02/04) or speciality for outpatient clinic attendance (SMR00), number of and British National Formulary (BNF) chapter and section code for prescriptions (PIS), and cause of death (Supplementary Table S.5). For A&E data only the number of attendances per year for each child was available. For hospitalisations, we also have access to outcomes from 1990 to account for past health. Previous research has identified differences in health service use (in primary care) among both mothers and CEC before the child entered care.[21] For A&E data past events are recorded from 2007 but for prescriptions past data was not available. For SMR02 and SMR04 admission reason was also recorded and will be used in research if the quality permits.

The descriptive analysis of the data in Table 3 shows that the proportion of CEC who have had at least one event recorded in any of the health data sources is higher compared to CGP for all data sets except for prescriptions. The biggest differences are evident for deaths, with CEC have 5.5 times higher mortality compared to CGP ($CEC_{rate}$ = 83.5, $CGP_{rate}$ = 15.3), and hospitalisations for maternity (CEC have 4.3 times higher rates in SMR02; $CEC_{rate}$ = 158.5, $CGP_{rate}$ = 36.6) and mental health



For peer review only - http://bmjopen.bmj.com/site/about/guidelines.xhtml

inpatient and day cases (CEC have 5.2 times higher rates in SMR04; $CEC_{rate}$ = 923.2, $CGP_{rate}$ = 179.2). CEC also experience substantially more health events (hospital visits, prescriptions) per child in all health data sets, with the biggest differences evident for SMR02, SMR04 and A&E attendances. A similar proportion of CEC and CGP have received at least one prescription during the study period, but the average number of prescriptions is higher for CEC, meaning that, on average, CEC received more prescriptions.

The next tables give the most common prescriptions (Table 4), outpatient clinic specialties (Table 5), and diagnosed condition for acute inpatient admissions (Table 6). (For coding schemas see Supplementary Table S.5.) The outcomes are ordered from largest to smallest by the total number of events for each health outcome and separately for the two cohorts. The resulting different ordering for the two cohorts is intentional and aims to highlight the very marked differences in health between the two cohorts. The tables clearly show that the reasons for contacting the health services are dissimilar between the two cohorts. For example, a higher proportion of the CEC cohort have had prescriptions for depression (CEC = 19.7%, CGP = 8.1%), psychiatric outpatient clinic attendances (CEC = 20.1%, CGP = 5.5%) and acute inpatient admissions due to mental and behavioural disorders (CEC = 2.2%, CGP = 0.3%). The proportion of CEC hospitalised due to injuries, drug poisoning and other external causes is also higher compared to CGP (for drug poisoning CEC = 6.1%, CGP = 0.9%). The differences between the two cohorts in age-standardised rates for these prescriptions and hospitalisations are similarly notable.

A higher proportion of care experienced young women have had outpatient obstetrics (CEC = 17.7%, CGP = 3.8%), gynaecology (CEC = 14.5%, CGP = 8.0%) and midwifery (CEC = 4.1%, CGP = 0.4%) clinic attendances, and have had an abortion (CEC = 7.1%, CGP = 3.8%). The age-standardised rates for these hospital attendances are also higher for the care experienced young women compared to the women in the general population. The proportion of care experienced young women who have been prescribed contraceptives is similar to that of young women in the general population (CEC = 42.5%, CGP = 41.7%), however the total number of contraceptive prescriptions and the age-standardized rates are higher for the women in the general population ($CEC_{rate}$ = 218.4, $CGP_{rate}$ = 349.0). Without additional data on the type of contraceptive prescribed (long-acting, e.g. implants, *versus* not long-acting, e.g. oral contraceptives) we cannot say what drives this difference. However, previous research suggests that women from more deprived areas are more likely to received long-acting reversible contraceptives,[22] and this could explain why the care experienced women in our cohort have fewer prescriptions compared to the women in the general population.

These initial results show substantial differences in health and health service use between the two cohorts. First, the proportion of children who have been in contact with the health services is higher for the care experienced cohort compared to the CGP. Second, CEC have more frequent contact with health services across all data sets. Third, many of the reasons for contacting health services are different for the two cohorts, with the CEC having more frequent contact related to mental, sexual and reproductive health. The level of contact with the health services for some causes (e.g. outpatient attendance for ear, nose and throat diseases) is similar for the two cohorts.

The CHiCS cohorts outlined here show substantial differences in the socioeconomic background and health between children who have experienced care and those who have not. They provide a unique opportunity for the first population-wide evidence on objective health outcomes for an understudied and vulnerable population of children. Next, our research will focus on explaining the differences in the health outcomes between the two cohorts of children using event history analysis on matched cohorts.



For peer review only - http://bmjopen.bmj.com/site/about/guidelines.xhtml

## Collaboration

To access the data, the authors applied for data access to the Public Benefit and Privacy Panel for Health and Social Care and to the Scottish Government's Statistics Public Benefit and Privacy Panel. After data access was approved by both organizations a data sharing agreement between the University of Glasgow and the Scottish Government was agreed. Due to the sensitivity of the data, it will not be made publicly available.

We encourage collaboration from other researchers, charities and public bodies interested in this research that fit with the original aims of this study. We also encourage collaborations to extend the follow-up period of both cohorts to 2020 and beyond to study the effects of national lock-downs on the health of young adults and care leavers.

## Ethics

Ethical approval was obtained from the University of Glasgow College of Medicine, Veterinary and Life Sciences Ethics Committee.

## Funding

This work was supported by the Economic and Social Research Council [grant number ES/T000120/1]. The data linkage and access to the National Safe Haven was facilitated by the Urban Big Data Centre [grant number ES/L011921/1]. MA, DB and AHL are also funded by the Medical Research Council (MC_UU_00022/2) and the Scottish Government Chief Scientist Office (SPHSU17).

## Acknowledgements

The authors would like to acknowledge the support of the eDRIS Team (Public Health Scotland) for their involvement in obtaining approvals, provisioning and linking data and the use of the secure analytical platform within the National Safe Haven. The National Records of Scotland undertook the indexing and provided the linkage key for this study. The authors would also like to acknowledge the Scottish Exchange of Data (ScotXed) for allowing us to access their data and the CHiCS project Advisory Group members for their engagement and feedback on the initial study results.

## Author contributions

MA and DB conceived the research idea and carried out most of the research and writing. CTBL and CM assisted in initial data analysis and provided feedback on the draft of this paper. AL and MH contributed to the research design and have provided important feedback throughout the project and writing of this paper.



For peer review only - http://bmjopen.bmj.com/site/about/guidelines.xhtml

## Competing interest

No competing interests declared.

For peer review only



For peer review only - http://bmjopen.bmj.com/site/about/guidelines.xhtml

## References

1. Meltzer H, Lader D, Corbin T, Goodman R, Ford T. The mental health of young people looked after by local authorities in Scotland. Office for National Statistics; 2004.

2. Ridley J, McCluskey S. Exploring the Perceptions of Young People in Care and Care Leavers of the Health needs. Scott J Resid Care. 2003;2(1):55–65.

3. Reilly C, Johnson DR, Ferguson K. Validation of the Massachusetts Youth Screening Instrument with a looked after population. Clin Child Psychol Psychiatry. 2018 Sep;1359104518799119.

4. McMahon AD, Elliott L, Macpherson LM, Sharpe KH, Connelly G, Milligan I, et al. Inequalities in the dental health needs and access to dental services among looked after children in Scotland: a population data linkage study. Arch Dis Child [Internet]. 2017; Available from: https://adc.bmj.com/content/early/2017/08/18/archdischild-2016-312389

5. Brown D, Allik M, Dundas R, Leyland AH. All-cause and cause-specific mortality in Scotland 1981-2011 by age, sex and deprivation: a population-based study. Eur J Public Health [Internet]. 2019 Feb; Available from: https://dx.doi.org/10.1093/eurpub/ckz010

6. Bywaters P, Scourfield J, Jones C, Sparks T, Elliott M, Hooper J, et al. Child welfare inequalities in the four nations of the UK. J Soc Work. 2018 Sep;1468017318793479.

7. Murray ET, Lacey R, Maughan B, Sacker A. Association of childhood out-of-home care status with all-cause mortality up to 42-years later: Office of National Statistics Longitudinal Study. BMC Public Health. 2020;20(1):735.

8. Viner RM, Taylor B. Adult health and social outcomes of children who have been in public care: population-based study. Pediatrics. 2005 Apr;115(4):894–9.

9. Martin A, Ford T, Goodman R, Meltzer H, Logan S. Physical illness in looked-after children: a cross-sectional study. Arch Dis Child. 2014;99(2):103–7.

10. Berlin M, Vinnerljung B, Hjern A. School performance in primary school and psychosocial problems in young adulthood among care leavers from long term foster care. Child Youth Serv Rev. 2011 Dec;33(12):2489–97.

11. Taussig HN, Harpin SB, Maguire SA. Suicidality among preadolescent maltreated children in foster care. Child Maltreat. 2014/02/24 ed. 2014 Feb;19(1):17–26.

12. Vinnerljung B, Hjern A, Lindblad F. Suicide attempts and severe psychiatric morbidity among former child welfare clients - a national cohort study. J Child Psychol Psychiatry. 2006 Jul;47(7):723–33.

13. Vinnerljung B, Ribe M. Mortality after care among young adult foster children in Sweden. Int J Soc Welf. 2001;10(3):164–73.

14. Katz LY, Au W, Singal D, Brownell M, Roos N, Martens PJ, et al. Suicide and suicide attempts in children and adolescents in the child welfare system. CMAJ Can Med Assoc J J Assoc Medicale Can. 2011 Nov;183(17):1977–81.



For peer review only - http://bmjopen.bmj.com/site/about/guidelines.xhtml

15. Hjern A, Vinnerljung B, Lindblad F. Avoidable mortality among child welfare recipients and intercountry adoptees: a national cohort study. J Epidemiol Community Health. 2004;58(5):412–7.

16. Rubin DM, Alessandrini EA, Feudtner C, Localio AR, Hadley T. Placement Changes and Emergency Department Visits in the First Year of Foster Care. Pediatrics. 2004 Sep;114(3):e354.

17. Craine N, Midgley C, Zou L, Evans H, Whitaker R, Lyons M. Elevated teenage conception risk amongst looked after children; a national audit. Public Health. 2014 Jul;128(7):668–70.

18. Scott S, Hattie R, Tannahill C. Looked After Children in Glasgow and Scotland: A Health Needs Assessment. ScotPHN; 2013.

19. Clark D, King A, Sharpe K, Connelly G, Elliott L, Macpherson LMD, et al. Linking routinely collected social work, education and health data to enable monitoring of the health and health care of school-aged children in state care ('looked after children') in Scotland: a national demonstration project. Public Health. 2017 Sep;150:101–11.

20. The Scottish Government. Pupil census: supplementary statistics [Internet]. 2021 [cited 2021 Jun 17]. Available from: https://www.gov.scot/publications/pupil-census-supplementary-statistics/

21. Simkiss DE, Spencer NJ, Stallard N, Thorogood M. Health service use in families where children enter public care: a nested case control study using the General Practice Research Database. BMC Health Serv Res. 2012;12(1):65.

22. Morgan CR, Liu H. The relationship between area deprivation and prescription of long-acting reversible contraception in women of reproductive age in Lothian, Scotland, UK. BMJ Sex Reprod Health. 2017;43(4):281–8.

23. SCIS SC of IS. Facts and Figures [Internet]. 2021 [cited 2021 Jun 16]. Available from: https://www.scis.org.uk/facts-and-figures/

For peer review only



For peer review only - http://bmjopen.bmj.com/site/about/guidelines.xhtml

## Tables

### *Table 1: Individual level socioeconomic profiles of CEC and CGP cohorts*

| | Children with birth records | | | | All children | | | |
|---|---|---|---|---|---|---|---|---|
| | CGP | | CEC | | CGP | | CEC | |
| | N | % | N | % | N | % | N | % |
| **Number (%) of children** | **571,702** | **88.0** | **12,250** | **88.6** | **649,771** | **100.0** | **13,831** | **100.0** |
| **Female** | 281,221 | 49.2 | 5,565 | 45.4 | 319,438 | 49.2 | 6,274 | 45.4 |
| **Male** | 290,481 | 50.8 | 6,685 | 54.6 | 330,333 | 50.8 | 7,557 | 54.6 |
| **Age in months** | | | | | | | | |
| *mean (SD)* | *130 (43.0)* | | *135 (42.2)* | | *131 (43.0)* | | *136 (42.3)* | |
| **Year of birth** | | | | | | | | |
| 1990-1995 | 160,644 | 28.1 | 4,114 | 33.6 | 184,923 | 28.5 | 4,830 | 34.9 |
| 1996-2000 | 234,120 | 41.0 | 4,862 | 39.7 | 265,453 | 40.9 | 5,413 | 39.1 |
| 2001-2004 | 176,938 | 30.9 | 3,274 | 26.7 | 199,395 | 30.7 | 3,588 | 25.9 |
| **For children with birth records:** | | | | | | | | |
| **Mothers' age at birth** | | | | | | | | |
| *mean (SD)* | *28.3 (5.7)* | | *24.5 (5.8)* | | | | | |
| Aged under 20 | 43,624 | 7.6 | 2,792 | 22.8 | | | | |
| Aged 20-24 | 105,653 | 18.5 | 4,066 | 33.2 | | | | |
| Aged 25-29 | 175,114 | 30.6 | 2,898 | 23.7 | | | | |
| Aged 30-34 | 167,634 | 29.3 | 1,664 | 13.6 | | | | |
| Aged 35-39 | 68,442 | 12.0 | 682 | 5.6 | | | | |
| Aged 40+ | 10,737 | 1.9 | 130 | 1.1 | | | | |
| *Missing* | *498* | *0.1* | *18* | *0.1* | | | | |
| **Parental employment status at birth** | | | | | | | | |
| *Parental (for births 1990-1995)* | | | | | | | | |
| Employee | 117,526 | 73.2 | 2,715 | 66.0 | | | | |
| Manager | 14,836 | 9.2 | 84 | 2.0 | | | | |
| Supervisor | 4,788 | 3.0 | 35 | 0.9 | | | | |
| Self-employed (with employees) | 4,919 | 3.1 | 27 | 0.7 | | | | |
| Self-employed (without employees) | 6,955 | 4.3 | 67 | 1.6 | | | | |
| Student/Unemployed/Not available | 11,620 | 7.2 | 1,186 | 28.8 | | | | |
| *Missing* | *0* | *0.0* | *0* | *0.0* | | | | |
| *Mother (for births 1996-2004)* | | | | | | | | |
| Employee | 271,711 | 66.1 | 3,138 | 38.6 | | | | |
| Manager | 28,201 | 6.9 | 58 | 0.7 | | | | |
| Supervisor | 9,315 | 2.3 | 39 | 0.5 | | | | |
| Self-employed (with employees) | 3,811 | 0.9 | 7 | 0.1 | | | | |
| Self-employed (without employees) | 7,558 | 1.8 | 33 | 0.4 | | | | |
| Student/Unemployed/Not available | 90,425 | 22.0 | 4,861 | 59.7 | | | | |
| *Missing* | *37* | 0.0 | *0* | 0.0 | | | | |
| *Father (for births 1996-2004)* | | | | | | | | |
| Employee | 274,748 | 66.8 | 4,467 | 54.9 | | | | |
| Manager | 44,524 | 10.8 | 130 | 1.6 | | | | |
| Supervisor | 12,791 | 3.1 | 61 | 0.7 | | | | |
| Self-employed (with employees) | 15,036 | 3.7 | 79 | 1.0 | | | | |
| Self-employed (without employees) | 24,631 | 6.0 | 285 | 3.5 | | | | |
| Student/Unemployed/Not available | 15,889 | 3.9 | 1,227 | 15.1 | | | | |
| *Missing* | *23,439* | *5.7* | *1,887* | *23.2* | | | | |



For peer review only - http://bmjopen.bmj.com/site/about/guidelines.xhtml

For peer review only

Table 2: Deprivation level of area of residence of CEC and CGP cohorts

| | All children with birth records | | | | All children with birth records | | | | All children | | | |
|---|---|---|---|---|---|---|---|---|---|---|---|---|
| | *Area of residence at birth* | | | | *Area of residence in 2009* | | | | *Area of residence in 2009* | | | |
| | **CGP** | | **CEC** | | **CGP** | | **CEC** | | **CGP** | | **CEC** | |
| | **N** | **%** | **N** | **%** | **N** | **%** | **N** | **%** | **N** | **%** | **N** | **%** |
| **Number (%) of children** | **571,702** | **88.0** | **12,250** | **88.6** | **571,702** | **88.0** | **12,250** | **88.6** | **649,771** | **100.0** | **13,831** | **100.0** |
| **Quintile of deprivation** | | | | | | | | | | | | |
| Q1 (most deprived) | 142,253 | 24.9 | 7,166 | 58.5 | 123,331 | 21.6 | 5,061 | 41.3 | 140,120 | 21.6 | 5,591 | 40.4 |
| Q2 | 116,146 | 20.3 | 2,650 | 21.6 | 112,764 | 19.7 | 2,636 | 21.5 | 125,764 | 19.4 | 2,914 | 21.1 |
| Q3 | 107,180 | 18.7 | 1,420 | 11.6 | 110,157 | 19.3 | 1,641 | 13.4 | 125,704 | 19.3 | 1,852 | 13.4 |
| Q4 | 103,610 | 18.1 | 694 | 5.7 | 112,379 | 19.7 | 1,116 | 9.1 | 129,566 | 19.9 | 1,268 | 9.2 |
| Q5 (least deprived) | 101,665 | 17.8 | 303 | 2.5 | 112,846 | 19.7 | 613 | 5.0 | 127,979 | 19.7 | 696 | 5.0 |
| *Missing* | *858* | *0.2* | *17* | *0.1* | *225* | *0.0* | *1,183* | *9.7* | *638* | 0.1 | *1,510* | 10.9 |



For peer review only - http://bmjopen.bmj.com/site/about/guidelines.xhtml

*Table 3: Main health outcomes for the two cohorts, 2009-2016*

| | Children with at least one event | | | | Mean per child | | Rate* | | Ratio of rates |
|---|---|---|---|---|---|---|---|---|---|
| | CGP | | CEC | | CGP | CEC | CGP | CEC | CEC:CGP |
| | N | %** | N | % | | | | | |
| **Total children** | 649,771 | 100 | 13,831 | 100 | | | | | |
| **Total female** | 319,438 | 49.2 | 6,274 | 45.4 | | | | | |
| Deaths | 746 | 0.1 | 78 | 0.6 | 0.11 | 0.56 | 15.3 | 83.5 | 5.48 |
| PIS | 603,628 | 92.9 | 12,597 | 91.1 | 28.32 | 34.71 | 3642.3 | 4446.0 | 1.22 |
| SMR00 | 382,590 | 58.9 | 9,427 | 68.2 | 6.30 | 8.09 | 469.7 | 736.1 | 1.57 |
| SMR01 | 179,551 | 27.6 | 5,404 | 39.1 | 2.22 | 2.60 | 94.0 | 150.5 | 1.60 |
| SMR02*** | 14,269 | 4.5 | 1,302 | 20.8 | 2.82 | 3.24 | 36.6 | 158.5 | 4.33 |
| SMR04 | 2,197 | 0.3 | 323 | 2.3 | 3.56 | 2.90 | 179.2 | 923.2 | 5.15 |
| A&E | 434,528 | 66.9 | 10,826 | 78.3 | 3.36 | 5.86 | 273.2 | 572.1 | 2.09 |

Note: SMR00 - Outpatient Attendance; SMR01 - General/ Acute Inpatient and Day Case; SMR02 - Maternity Inpatient and Day Case; SMR04 - Mental Health Inpatient and Day Case
*Age standardized for ages 0-24 using the 2013 European standard population per 1000 person-years (PY). For deaths and SMR04, rates are shown per 100,000 PY.
**Percent from cohort.
***Calculated for female members of the cohort.



For peer review only - http://bmjopen.bmj.com/site/about/guidelines.xhtml

*Table 4: PIS – Prescribing Information System*

| CGP | | | | | |
|---|---|---|---|---|---|
| **BNF Chapter/Section** | **Prescriptions** | | **Children** | | |
| | **N** | **%** | **N** | **%** | **Rate*** |
| 3 - Respiratory system | 3,350,333 | 19.6 | 334,231 | 51.4 | 689.1 |
| 301 - Bronchodilators | 1,164,625 | 6.8 | 130,986 | 20.2 | 241.5 |
| 304 - Antihistamines | 1,108,966 | 6.5 | 236,979 | 36.5 | 215.6 |
| 302 - Corticosteroids | 750,694 | 4.4 | 76,544 | 11.8 | 157.1 |
| 13 - Skin | 3,260,944 | 19.1 | 456,524 | 70.3 | 690.9 |
| 4 - Central Nervous System | 2,301,960 | 13.5 | 327,896 | 50.5 | 530.5 |
| 407 - Analgesics | 1,020,520 | 6.0 | 279,953 | 43.1 | 245.1 |
| 403 - Antidepressant | 398,369 | 2.33 | 52,768 | 8.1 | 114.4 |
| 404 - CNS and ADHD | 273,164 | 1.6 | 7548 | 1.2 | 41.16 |
| 5 - Infections | 2,234,038 | 13.1 | 470,671 | 72.4 | 487.8 |
| 501 - Antibacterial drugs | 2,055,439 | 12.0 | 454,724 | 70.0 | 444.7 |
| 703 - Contraceptives** | 779,348 | 4.6 | 133,343 | 41.7 | 349.0 |
| 1001 - Drugs used in rheumatic diseases & gout | 638,973 | 3.7 | 258,205 | 39.7 | 132.6 |
| 601 - Diabetes drugs | 592,741 | 3.5 | 5,396 | 0.8 | 105.5 |

| CEC | | | | | |
|---|---|---|---|---|---|
| **BNF Chapter/Section** | **Prescriptions** | | **Children** | | |
| | **N** | **%** | **N** | **%** | **Rate*** |
| 4 - Central Nervous System | 116,988 | 26.8 | 9,215 | 66.6 | 1265.2 |
| 407 - Analgesics | 31,394 | 7.2 | 7,570 | 54.7 | 393.4 |
| 404 - CNS and ADHD | 28,455 | 6.5 | 794 | 5.7 | 224.3 |
| 403 - Antidepressant | 20,922 | 4.8 | 2,723 | 19.7 | 260.2 |
| 3 - Respiratory system | 71,972 | 16.5 | 7,537 | 54.5 | 713.4 |
| 301 - Bronchodilators | 27,163 | 6.2 | 2,897 | 20.9 | 265.5 |
| 302 - Corticosteroids | 16,881 | 3.9 | 1,625 | 11.7 | 169.9 |
| 304 - Antihistamines | 18,663 | 4.3 | 4,779 | 34.6 | 169.5 |
| 13 - Skin | 62,960 | 14.4 | 9,707 | 70.2 | 621.5 |
| 5 - Infections | 45,127 | 10.3 | 9,520 | 68.8 | 448.8 |
| 501 - Antibacterial drugs | 41,721 | 9.5 | 9,178 | 66.4 | 411.0 |
| 1001 - Drugs used in rheumatic diseases & gout | 15,104 | 3.5 | 6,041 | 43.7 | 146.3 |
| 601 - Diabetes drugs | 14,391 | 3.3 | 139 | 1.0 | 124.9 |
| 703 - Contraceptives** | 11,552 | 2.6 | 2,685 | 42.8 | 218.4 |

Note: BNF sections are indented.
*Age standardized for age groups 0-24 using the 2013 European standard population per 1000 person-years.
**For contraceptives, the percent of children and the age-standardized rate are calculated for the female members of the cohort.

For peer review only

*Table 5: SMR00 – Outpatient Attendances*

| CGP | | | | | |
|---|---|---|---|---|---|
| **Clinic specialty** | **Attendances** | | **Children** | | |
| | **N** | **%** | **N** | **%** | **Rate*** |
| D5 - Orthodontics | 492,407 | 20.4 | 51,950 | 8.0 | 67.5 |
| C8 - Trauma & Orthopaedic Surgery | 318,094 | 13.2 | 121,722 | 18.7 | 58.8 |
| AF - Paediatrics | 233,131 | 9.7 | 66,860 | 10.3 | 49.3 |
| A7 - Dermatology | 203,359 | 8.4 | 61,886 | 9.5 | 40.3 |
| Psychiatry (all) | 195,907 | 8.1 | 35,802 | 5.5 | 32.6 |
| C5 - Ear, Nose & Throat | 133,917 | 5.6 | 63,502 | 9.8 | 32.2 |
| Dentistry (all) | 104,934 | 4.4 | 24,581 | 3.8 | 22.9 |
| C7 - Ophthalmology | 80,998 | 3.4 | 32,000 | 4.9 | 21.7 |
| A9 - Gastroenterology | 47,755 | 2.0 | 14,737 | 2.3 | 10.8 |
| Obstetrics** (all) | 47,015 | 2.0 | 12,135 | 3.8 | 26.2 |
| AH - Neurology | 46,675 | 1.9 | 17,394 | 2.7 | 9.4 |
| F2 – Gynaecology** | 45,324 | 1.9 | 25,508 | 8.0 | 23.6 |
| C9 - Plastic Surgery | 44,880 | 1.9 | 16,511 | 2.5 | 8.8 |
| T2 – Midwifery** | 20,510 | 0.9 | 2,561 | 0.4 | 11.3 |
| G5 - Learning Disability (Mental Handicap) | 5,033 | 0.2 | 1,026 | 0.2 | 1.4 |

| CEC | | | | | |
|---|---|---|---|---|---|
| **Clinic specialty** | **Attendances** | | **Children** | | |
| | **N** | **%** | **N** | **%** | **Rate*** |
| Psychiatry (all) | 20,170 | 26.5 | 2,781 | 20.1 | 163.2 |
| C8 - Trauma & Orthopaedic Surgery | 7,507 | 9.8 | 2,805 | 20.3 | 68.4 |
| AF - Paediatrics | 7,015 | 9.2 | 2,034 | 14.7 | 79.7 |
| D5 - Orthodontics | 6,180 | 8.1 | 851 | 6.2 | 42.2 |
| Obstetrics** (all) | 5,868 | 7.7 | 1,113 | 17.7 | 131.6 |
| C5 - Ear, Nose & Throat | 3,088 | 4.1 | 1,452 | 10.5 | 38.0 |
| Dentistry (all) | 2,957 | 3.9 | 797 | 5.8 | 30.8 |
| T2 – Midwifery** | 2,694 | 3.5 | 255 | 4.1 | 65.4 |
| A7 - Dermatology | 2,602 | 3.4 | 858 | 6.2 | 23.5 |
| G5 - Learning Disability (Mental Handicap) | 2,134 | 2.8 | 195 | 1.4 | 29.9 |
| C7 - Ophthalmology | 2,067 | 2.7 | 869 | 6.3 | 36.4 |
| F2 – Gynaecology** | 1,844 | 2.4 | 908 | 14.5 | 44.1 |
| AH - Neurology | 1,593 | 2.1 | 555 | 4.0 | 15.0 |
| C9 - Plastic Surgery | 1,070 | 1.4 | 393 | 2.8 | 11.0 |
| A9 - Gastroenterology | 725 | 1.0 | 273 | 2.0 | 6.8 |

*Age standardized for age groups 0-24 using the 2013 European standard population per 1000 person-years.

**For obstetrics, gynaecology and midwifery the percent of children and the age-standardised rate are calculated for the female members of the cohort.

For peer review only



For peer review only - http://bmjopen.bmj.com/site/about/guidelines.xhtml

*Table 6: SMR01 – General/Acute Inpatient and Day Cases by ICD-10 chapters*

| CGP | | | | | |
|---|---|---|---|---|---|
| Main diagnosis | Attendances | | Children | | |
| | N | % | N | % | Rate* |
| Diseases of the digestive system | 61,704 | 15.5 | 41,483 | 6.4 | 17.0 |
| Dental caries (K02) | 14,084 | 3.5 | 13,301 | 2.0 | 7.0 |
| Injury, poisoning and other consequences of external causes | 55,038 | 13.8 | 42,002 | 6.5 | 12.0 |
| Other injury | 28,726 | 7.2 | 22,899 | 3.5 | 6.3 |
| Head injury | 11,095 | 2.8 | 9,745 | 1.5 | 2.7 |
| Drug poisoning | 9,251 | 2.3 | 6,129 | 0.9 | 1.8 |
| Hand injury | 5,966 | 1.5 | 5,435 | 0.8 | 1.3 |
| Symptoms, signs and abnormal clinical and laboratory findings | 50,192 | 12.6 | 33,271 | 5.1 | 12.0 |
| Diseases of the respiratory system | 39,226 | 9.8 | 25,628 | 3.9 | 10.5 |
| Asthma | 9,172 | 2.3 | 7,562 | 1.2 | 2.7 |
| Diseases of the genitourinary system | 25,776 | 6.5 | 16,600 | 2.6 | 5.4 |
| Diseases of the musculoskeletal system and connective tissue | 23,431 | 5.9 | 12,365 | 1.9 | 4.5 |
| Neoplasms | 21,800 | 5.5 | 5,340 | 0.8 | 4.9 |
| Examination, observation, etc | 19,259 | 4.8 | 12,918 | 2.0 | 4.4 |
| Obstetric** | 15,099 | 3.8 | 13,089 | 4.1 | 6.9 |
| Medical abortion (O04)** | 13,574 | 3.4 | 12,107 | 3.8 | 6.1 |
| Endocrine, nutritional and metabolic diseases | 13,910 | 3.5 | 3,978 | 0.6 | 2.6 |
| Mental and behavioural disorders | 2,646 | 0.664 | 2,207 | 0.3 | 0.5 |

| CEC | | | | | |
|---|---|---|---|---|---|
| Main diagnosis | Attendances | | Children | | |
| | N | % | N | % | Rate* |
| Injury, poisoning and other consequences of external causes | 3,900 | 27.7 | 2,198 | 15.9 | 36.2 |
| Drug poisoning | 1,671 | 11.9 | 849 | 6.1 | 13.0 |
| Other injury | 1,262 | 9.0 | 895 | 6.5 | 10.7 |
| Head injury | 626 | 4.5 | 514 | 3.7 | 5.2 |
| Hand Injury | 341 | 2.4 | 298 | 2.2 | 2.9 |
| Symptoms, signs and abnormal clinical and laboratory findings | 1,811 | 12.9 | 1,059 | 7.7 | 17.3 |
| Diseases of the digestive system | 1,626 | 11.6 | 1,158 | 8.4 | 27.2 |
| Dental caries (K02) | 631 | 4.5 | 590 | 4.3 | 17.7 |
| Diseases of the respiratory system | 1,108 | 7.9 | 618 | 4.5 | 12.4 |
| Asthma | 317 | 2.3 | 116 | 0.8 | 2.9 |
| Diseases of the genitourinary system | 867 | 6.2 | 463 | 3.3 | 7.3 |
| Obstetric** | 702 | 5.0 | 539 | 8.6 | 15.5 |
| Medical abortion (O04)** | 538 | 3.8 | 447 | 7.1 | 11.3 |
| Endocrine, nutritional and metabolic diseases | 604 | 4.3 | 114 | 0.8 | 5.7 |
| Examination, observation, etc | 566 | 4.0 | 426 | 3.1 | 5.1 |
| Diseases of the musculoskeletal system and connective tissue | 401 | 2.9 | 234 | 1.7 | 5.9 |
| Mental and behavioural disorders | 399 | 2.8 | 310 | 2.2 | 3.0 |
| Neoplasms | 168 | 1.195 | 80 | 0.6 | 1.4 |

Note: Subgroups within ICD-10 chapters are indented.

*Age standardized for age groups 0-24 using the 2013 European standard population per 1000 person-years.

**For obstetrics and medical abortion, the percent of children and the age-standardised rate are calculated for the female members of the cohort.

For peer review only



For peer review only - http://bmjopen.bmj.com/site/about/guidelines.xhtml

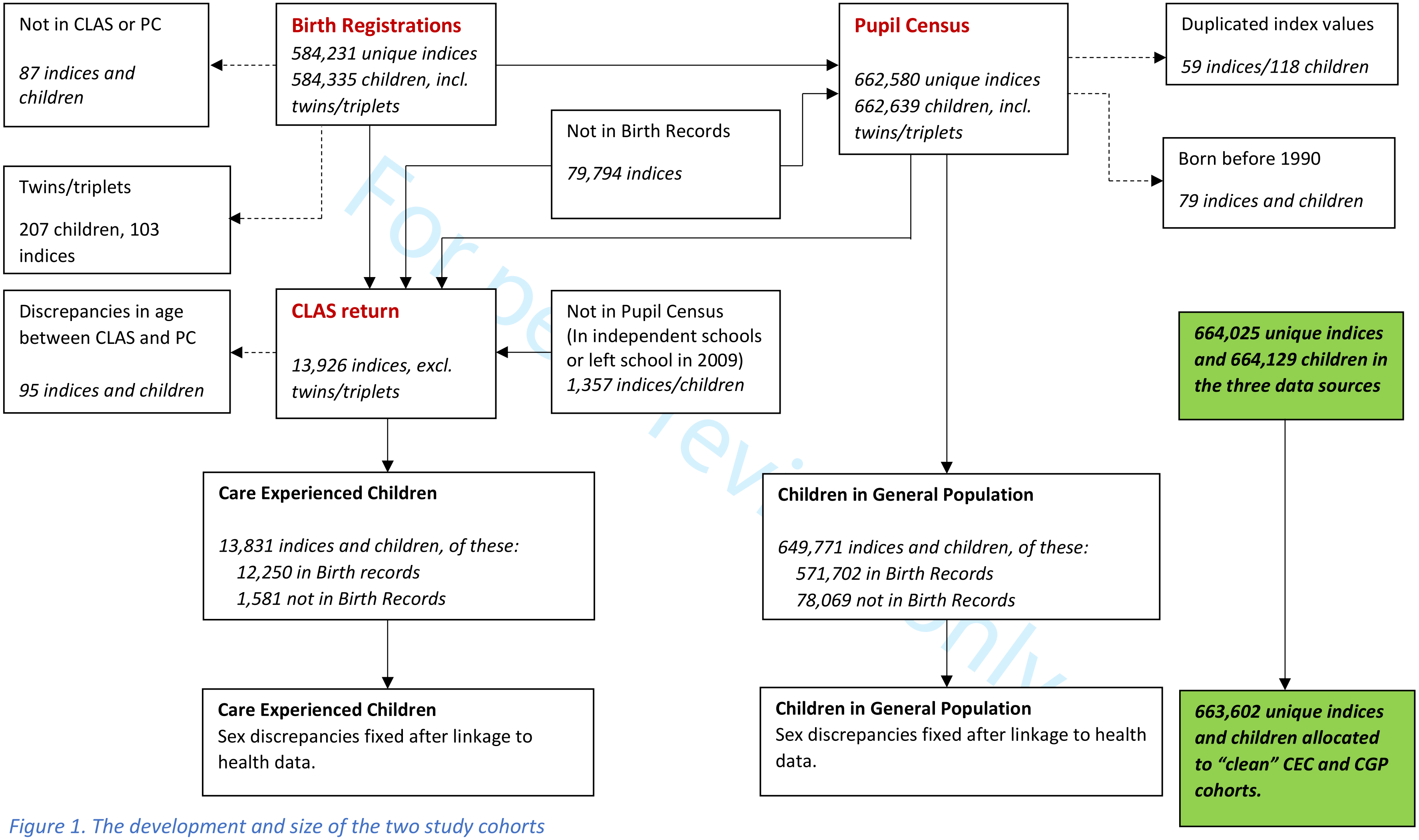


*Figure 1. The development and size of the two study cohorts*

Note: Dashed arrows indicate children who were removed from the cohorts during data cleaning.

## Supplement

*Table S.1: Urban-Rural Classification and Health Board of residence of CEC and CGP cohorts*

| | All children with birth records *Area of residence at birth* | | | | All children with birth records *Area of residence in 2009* | | | | All children *Area of residence in 2009* | | | |
|---|---|---|---|---|---|---|---|---|---|---|---|---|
| | CGP | | CEC | | CGP | | CEC | | CGP | | CEC | |
| | N | % | N | % | N | % | N | % | N | % | N | % |
| **Number (%) of children** | **571,702** | **88.0** | **12,250** | **88.6** | **571,702** | **88.0** | **12,250** | **88.6** | **649,771** | **100.0** | **13,831** | **100.0** |
| **Urban-Rural classification** | | | | | | | | | | | | |
| Large urban areas | 220,713 | 38.6 | 6,164 | 50.3 | 199,694 | 34.9 | 4,853 | 39.6 | 226,405 | 34.8 | 5,349 | 38.7 |
| Other urban areas | 172,673 | 30.2 | 3,580 | 29.2 | 188,845 | 33.0 | 3,509 | 28.6 | 209,340 | 32.2 | 3,893 | 28.1 |
| Accessible small towns | 58,649 | 10.3 | 1,012 | 8.3 | 52,500 | 9.2 | 861 | 7.0 | 59,155 | 9.1 | 944 | 6.8 |
| Remote small towns | 15,772 | 2.8 | 363 | 3.0 | 22,037 | 3.9 | 460 | 3.8 | 25,424 | 3.9 | 505 | 3.7 |
| Accessible rural | 74,024 | 12.9 | 823 | 6.7 | 72,390 | 12.7 | 927 | 7.6 | 83,801 | 12.9 | 1,068 | 7.7 |
| Remote rural | 29,013 | 5.1 | 291 | 2.4 | 36,011 | 6.3 | 457 | 3.7 | 45,008 | 6.9 | 562 | 4.1 |
| *Missing* | *858* | *0.2* | *17* | *0.1* | *225* | *0.0* | *1,183* | 9.7 | *638* | 0.1 | *1,510* | 10.9 |
| **Health Boards*** | | | | | | | | | | | | |
| Ayrshire & Clyde | 46,869 | 8.2 | 1,356 | 11.1 | - | - | - | - | - | - | - | - |
| Ayrshire & Arran | 41,008 | 7.2 | 952 | 7.8 | 43,278 | 7.6 | 958 | 7.8 | 47,621 | 7.3 | 1,072 | 7.8 |
| Borders | 10,681 | 1.9 | 131 | 1.1 | 12,031 | 2.1 | 144 | 1.2 | 14,663 | 2.3 | 165 | 1.2 |
| Dumfries & Galloway | 15,581 | 2.7 | 261 | 2.1 | 16,037 | 2.8 | 263 | 2.1 | 19,064 | 2.9 | 304 | 2.2 |
| Fife | 39,279 | 6.9 | 640 | 5.2 | 41,013 | 7.2 | 712 | 5.8 | 45,907 | 7.1 | 796 | 5.8 |
| Forth Valley | 31,866 | 5.6 | 716 | 5.8 | 33,848 | 5.9 | 676 | 5.5 | 37,965 | 5.8 | 753 | 5.4 |
| Grampian | 54,813 | 9.6 | 1,157 | 9.4 | 55,016 | 9.6 | 1,013 | 8.3 | 65,622 | 10.1 | 1,128 | 8.2 |
| Greater Glasgow (&Clyde) | 103,106 | 18.0 | 2,889 | 23.6 | 132,673 | 23.2 | 3,242 | 26.5 | 148,650 | 22.9 | 3,577 | 25.9 |
| Highland | 24,089 | 4.2 | 390 | 3.2 | 34,323 | 6.0 | 552 | 4.5 | 41,643 | 6.4 | 636 | 4.6 |
| Lanarkshire | 70,439 | 12.3 | 935 | 7.6 | 73,274 | 12.8 | 1,041 | 8.5 | 89,242 | 13.7 | 1,142 | 8.3 |
| Lothian | 83,929 | 14.7 | 1,833 | 15.0 | 79,506 | 13.9 | 1,595 | 13.0 | 90,757 | 14.0 | 1,755 | 12.7 |
| Orkney | 2,145 | 0.4 | 21 | 0.2 | 2,206 | 0.4 | 23 | 0.2 | 2,709 | 0.4 | 29 | 0.2 |
| Shetland | 2,789 | 0.5 | 27 | 0.2 | 2,754 | 0.5 | 24 | 0.2 | 3,283 | 0.5 | 27 | 0.2 |
| Tayside | 41,514 | 7.3 | 893 | 7.3 | 42,602 | 7.5 | 787 | 6.4 | 48,465 | 7.5 | 891 | 6.4 |
| Western Isles | 2,736 | 0.5 | 32 | 0.3 | 2,916 | 0.5 | 37 | 0.3 | 3,557 | 0.5 | 46 | 0.3 |
| *Missing* | *858* | *0.2* | *17* | *0.1* | *225* | *0.0* | *1,183* | *9.7* | *638* | 0.1 | *1,510* | 10.9 |

*Health Board at birth based on 1995 Health Boards. Health Board in 2009 based on 2006 Health Boards. In 2006, NHS Argyll and Clyde responsibilities were shared between NHS Highland and NHS Greater Glasgow.



For peer review only - http://bmjopen.bmj.com/site/about/guidelines.xhtml

*Table S.2: Residential stability of datazone between birth and 2009*

| | CGP with birth records | | CEC with birth records | |
|---|---|---|---|---|
| | **N** | **%** | **N** | **%** |
| | **571,702** | | **12,250** | |
| Same data zone at birth and in 2009 | 215,308 | 37.7 | 1,523 | 12.4 |
| Moved data zone between birth and 2009 | 355,324 | 62.2 | 9,527 | 77.8 |
| *Missing** | *1,070* | *0.2* | *1,200* | *9.8* |

*Of data zones missing in the CGP cohort: 858 are missing at birth, 225 are missing in 2009 and 13 are missing in both periods. Of data zones missing in the CEC cohort: 17 are missing at birth and 1183 are missing in 2009.

*Table S.3: Comparison of CEC cohort to national rates of children looked after by sex and age*

| | CEC cohort: Children on 2009/10 CLAS return and 2009 PC | | National rates of Children Looked After* | | | | CEC cohort as % from national rates of Children Looked After |
|---|---|---|---|---|---|---|---|
| | N | % | A) Looked after at 31 March 2009 | B) Starting to be looked after 01/08/2009 - 31/07/2010 | A + B | % | |
| Female | 6,274 | 45.4 | 6,912 | 2,288 | 9,200 | 45.2 | 68.2 |
| Male | 7,557 | 54.6 | 8,375 | 2,571 | 10,946 | 54.8 | 69.0 |
| | | | | | | | |
| Age (at 31st July 2009) | | | | | | | |
| 0-4 | 336 | 2.4 | 3,157 | 1,796 | 4,953 | 20.7 | 6.8 |
| 5 to 11 | 6,670 | 48.2 | 5,510 | 1,463 | 6,973 | 36 | 95.7 |
| 12 to 15 | 5,845 | 42.3 | 5,017 | 1,555 | 6,572 | 32.8 | 88.9 |
| 16 & 17 | 956 | 6.9 | 1,457 | 45 | 1,502 | 9.5 | 63.6 |
| 18+ | 24 | 0.2 | 146 | 0 | 146 | 1 | 16.4 |
| | | | | | | | |
| Total | 13,831 | 100 | 15,287 | 4,859 | 20,146 | 100 | 68.7 |

*Source: https://www.gov.scot/publications/statistics-publication-notice-health-care-series-children-looked-statistics-2009-10/pages/1/



For peer review only - http://bmjopen.bmj.com/site/about/guidelines.xhtml

*Table S.4: Comparison of all children in study (CEC and CGP) to Scotland's 2011 census population, age 0-19 years*

| | All children in study | | Scotland's census 2011** | | |
|---|---|---|---|---|---|
| | **N** | **%** | **N** | **%** | |
| **All children in study (CEC and CGP), at 31 July 2009*** | **663,602** | | | | |
| **All children in Scotland at 2011 census** | | | **1,184,879** | | |
| | | | | | *% of census population* |
| **Female** | 325,712 | 49.1 | 579,949 | 48.9 | *56.2* |
| **Male** | 337,890 | 50.9 | 604,930 | 51.1 | *55.9* |
| **Age** | | | | | |
| 0-4 | 20,957 | 3.2 | 292,821 | 24.7 | *7.2* |
| 5 to 11 | 364,271 | 54.9 | 381,453 | 32.2 | *95.5* |
| 12 to 15 | 226,091 | 34.1 | 242,057 | 20.4 | *93.4* |
| 16 & 17 | 51,964 | 7.8 | 126,266 | 10.7 | *41.2* |
| 18 & 19 | 319 | 0.0 | 142,282 | 12.0 | *0.2* |

*Includes only those children in publicly-funded schools.

**Source: Scotland's Census 2011. Table DC1117SC – Age by sex, All people



For peer review only - http://bmjopen.bmj.com/site/about/guidelines.xhtml

*Table S.5: Health outcomes of interest*

| Data set | Prior health | Outcome measures | Total events 2009-2016 *N* Valid | % Valid* |
|---|---|---|---|---|
| Death Records | NA | Number of deaths | | |
| | | Cause of death | 824 | 100 |
| PIS - Prescribing Information System | No | Number of prescriptions | | |
| | | BNF chapter/section | 17,533,253 | 99.3 |
| SMR00 - Outpatient Attendance | Yes | Number of admissions | | |
| | | Speciality | 2,487,792 | 100 |
| SMR01 - General/ Acute Inpatient and Day Case | Yes | Number of admissions | | |
| | | Condition diagnosed | 412,616 | 100 |
| SMR02 - Maternity Inpatient and Day Case | Yes | Number of admissions | | |
| | | Condition diagnosed | 44,447 | 100 |
| | | *Admission reason* | | 100 |
| SMR04 - Mental Health Inpatient and Day Case | Yes | Number of admissions | | |
| | | Condition diagnosed | 8,774 | 99.9 |
| | | *Admission reason* | | 83.8 |
| A&E - Accidents and Emergencies | Yes | Number of attendances per year | 1,523,815 | NA |

*Percent with valid code for BNF chapter/section, diagnosed condition or admission reason from total recorded events. Not applicable for A&E attendances.

For peer review only

*Table S.6: Coding schemas for SMR00 and SMR01*

| Label used in tables | Codes |
|---|---|
| **Clinic specialty SMR00** | **Specialty/Discipline Codes included*** |
| Psychiatry | G1, G1A, G2, G21, G22, G3, G4 |
| Dentistry | D6, D61-D63, D8, DC, DE, DD |
| Obstetrics | E11, F3, F31, F32 |
| | |
| **Main condition SMR01** | **ICD-10 codes included** |
| Diseases of the digestive system | K00-K93 |
| Injury, poisoning and certain other consequences of external causes | S00-T98 |
| Other injury | S10-S59, S70-T35, T51-T98 |
| Head injury | S00-S09 |
| Drug poisoning | T36-T50 |
| Hand injury | S60-S69 |
| | |
| Symptoms, signs and abnormal clinical and laboratory findings | R00-R99 |
| Diseases of the respiratory system | J00-J99 |
| Asthma | =J45-J46 |
| | |
| Diseases of the genitourinary system | N00-N99 |
| | |
| Diseases of the musculoskeletal system and connective tissue | M00-M99 |
| Neoplasms | C00-D48 |
| Examination, observation, etc | Z00-Z99 |
| Obstetric | O00-O99 |
| | |
| Endocrine, nutritional and metabolic diseases | E00-E90 |
| Mental and behavioural disorders | F00-F99 |

*Clinic specialty codes are described in the SMR00 crib sheet https://www.ndc.scot.nhs.uk/Data-Dictionary/SMR-Crib-Sheets/SMR00_CRIB_07012021.pdf



For peer review only

For peer review only - http://bmjopen.bmj.com/site/about/guidelines.xhtml

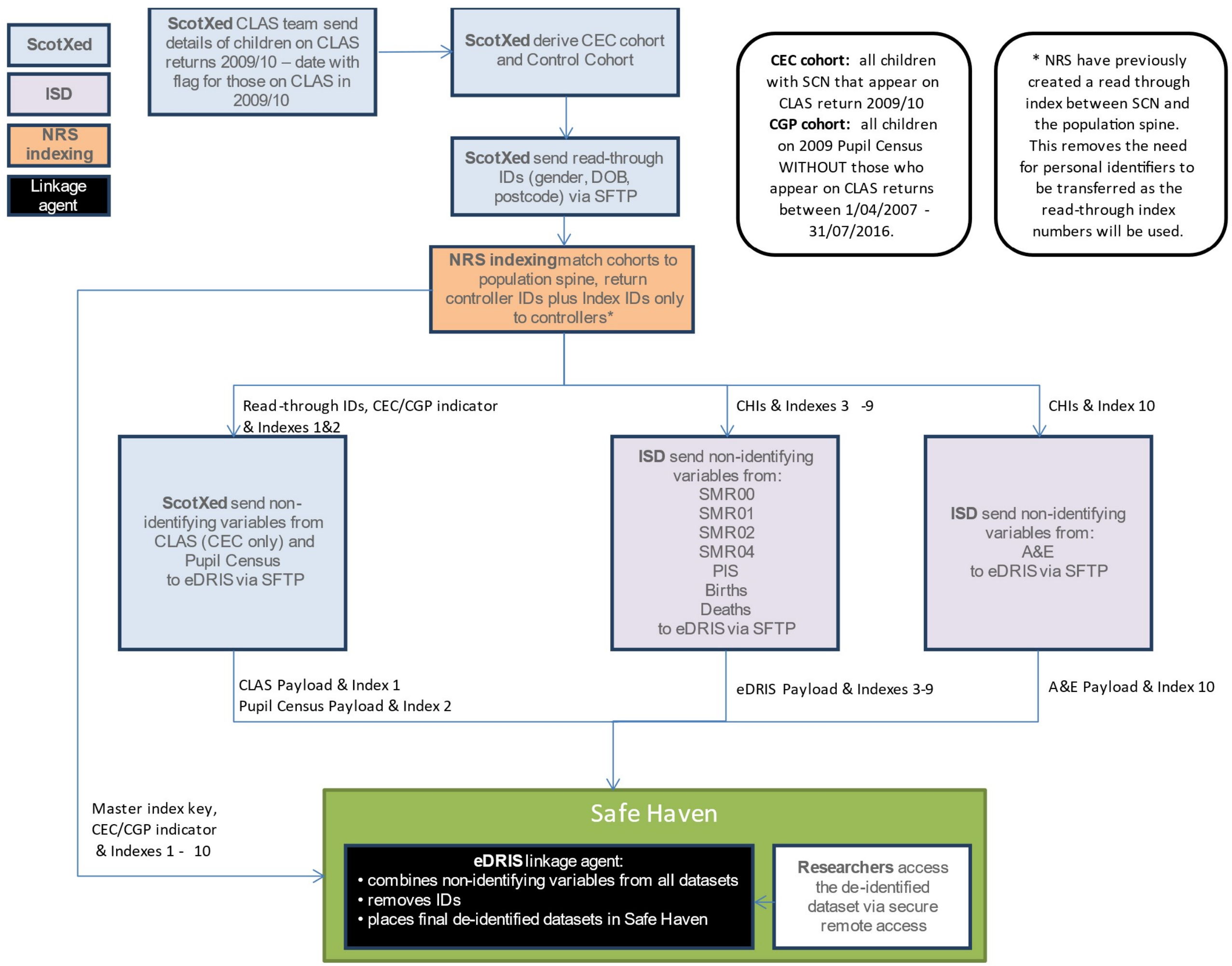


*Figure S.1. Linkage diagram for the CHiCS project*

CEC – care experienced children; CGP – children in the general population; SFTP – secure file transfer protocol; ScotXed – Scottish Exchange of Data; NRS – National Records of Scotland; ISD – Information Services Division (part of NHS); eDRIS – electronic Data Research and Innovation Service; SCN – Scottish Candidate Number



For peer review only - http://bmjopen.bmj.com/site/about/guidelines.xhtml

BMJ Open

# Cohort profile: The "Children's Health in Care in Scotland" (CHiCS) study – a longitudinal dataset to compare health outcomes for care experienced children and general population children.

| | |
|---|---|
| Journal: | *BMJ Open* |
| Manuscript ID | bmjopen-2021-054664.R1 |
| Article Type: | Cohort profile |
| Date Submitted by the Author: | 16-Aug-2021 |
| Complete List of Authors: | Allik, Mirjam; University of Glasgow, MRC/SCO Social and Public Health Sciences Unit<br>Brown, Denise ; University of Glasgow, MRC/CSO Social and Public Health Sciences Unit<br>Taylor Browne Luka, Courtney; University of Glasgow, School of Psychology<br>MacIntyre, Cecilia; Scottish Government, Data for Research Unit<br>Leyland, Alastair; University of Glasgow, MRC/CSO Social and Public Health Sciences Unit<br>Henderson, Marion; University of Strathclyde |
| <b>Primary Subject Heading</b>: | Epidemiology |
| Secondary Subject Heading: | Mental health, Obstetrics and gynaecology, Paediatrics, Public health, Sexual health |
| Keywords: | EPIDEMIOLOGY, Community child health < PAEDIATRICS, PUBLIC HEALTH, SOCIAL MEDICINE |
| | |

SCHOLARONE™
Manuscripts

I, the Submitting Author has the right to grant and does grant on behalf of all authors of the Work (as defined in the below author licence), an exclusive licence and/or a non-exclusive licence for contributions from authors who are: i) UK Crown employees; ii) where BMJ has agreed a CC-BY licence shall apply, and/or iii) in accordance with the terms applicable for US Federal Government officers or employees acting as part of their official duties; on a worldwide, perpetual, irrevocable, royalty-free basis to BMJ Publishing Group Ltd ("BMJ") its licensees and where the relevant Journal is co-owned by BMJ to the co-owners of the Journal, to publish the Work in this journal and any other BMJ products and to exploit all rights, as set out in our licence.

The Submitting Author accepts and understands that any supply made under these terms is made by BMJ to the Submitting Author unless you are acting as an employee on behalf of your employer or a postgraduate student of an affiliated institution which is paying any applicable article publishing charge ("APC") for Open Access articles. Where the Submitting Author wishes to make the Work available on an Open Access basis (and intends to pay the relevant APC), the terms of reuse of such Open Access shall be governed by a Creative Commons licence – details of these licences and which Creative Commons licence will apply to this Work are set out in our licence referred to above.

Other than as permitted in any relevant BMJ Author's Self Archiving Policies, I confirm this Work has not been accepted for publication elsewhere, is not being considered for publication elsewhere and does not duplicate material already published. I confirm all authors consent to publication of this Work and authorise the granting of this licence.

For peer review only

# Cohort profile: The "Children's Health in Care in Scotland" (CHiCS) study – a longitudinal dataset to compare health outcomes for care experienced children and general population children.

Mirjam Allik[1*], Denise Brown[1], Courtney Taylor Browne Lūka[2], Cecilia Macintyre[3], Alastair H Leyland[1], Marion Henderson[4]

[*] Corresponding author, email: mirjam.allik@glasgow.ac.uk

[1]MRC/CSO SPHSU
University of Glasgow
Berkeley Square
99 Berkeley Street
Glasgow G3 7HR

[2]School of Psychology
University of Glasgow
62 Hillhead Street
Glasgow G12 8QB

[3]Data for Research Unit
Statistics and Data Access Division
Scottish Government
1-F South, Victoria Quay, Edinburgh, EH6 6QQ

[4]School Of Social Work and Social Policy
University of Strathclyde
Lord Hope Building
141 St James Road
Glasgow
G4 0LT

Word count: ~4100

Keywords: care experienced children, looked after children, health inequalities, administrative data, data linkage

For peer review only



For peer review only - http://bmjopen.bmj.com/site/about/guidelines.xhtml

## Abstract

**Purpose:** The Children's Health in Care in Scotland Cohorts were set up to provide first population-wide evidence on the health outcomes of care experienced children (CEC) compared to children in the general population (CGP). To date, there are no data on how objective health outcomes, mortality and pregnancies for CEC are different from CGP in Scotland.

**Participants:** The CEC cohort includes school-aged children who were on the 2009/2010 Scottish Government's Children Looked After Statistics (CLAS) return and on the 2009 Pupil Census (PC). The children in the general population cohort includes those who were on the 2009 PC and not on any of the CLAS returns between April 1st, 2007 – July 31st, 2016.

**Findings to date:** Data on a variety of health outcomes, including mortality, prescriptions, hospitalisations, pregnancies, and Accident and Emergency (A&E) attendances, were obtained for the period August 1st, 2009 – July 31st, 2016 for both cohorts. Data on socioeconomic status (SES) for both cohorts were available from the Birth Registrations and a small area deprivation measure was available from the PC. CEC have, on average, lower SES at birth and live in areas of higher deprivation compared to CGP. A higher proportion of CEC have recorded events across all health data sets and they experienced higher average rates of mortality, prescriptions, and hospitalisations during the study period. The reasons for contacting health services vary between cohorts.

**Future plans:** Age-standardized rates for the two cohorts by sex and area deprivation will be calculated to provide evidence on population-wide prevalence of main causes of death, reasons for hospitalisation and types of prescription. Event history analysis will be used on matched cohorts to investigate the impact of placement histories and socioeconomic factors on health.


For peer review only



For peer review only - http://bmjopen.bmj.com/site/about/guidelines.xhtml

## Article Summary

## Strengths and limitations of this study

- The CHiCS project is the first population-wide longitudinal data collection in the UK that links administrative data on social care, births, deaths, hospitalisations and prescriptions to compare the health of care experienced children (CEC) to children in the general population (CGP).
- The study looks at a wide range of health outcomes, including in- and outpatient hospitalisations, prescriptions, accidents & emergency attendances, pregnancies and mortality.
- CEC had higher average rates of mortality, prescriptions, and hospitalisations during the study period compared to CGP, and the differences in health between the two cohorts are most notable for mental, sexual and reproductive health.
- The weaknesses of the study include errors in the individual data sets and in data linkage (affecting about 3% of the study population), and the exclusion of children who were not in school or were in independent schools (approximately 4% of all school-aged children).

For peer review only



For peer review only - http://bmjopen.bmj.com/site/about/guidelines.xhtml

## Introduction

There is very little quantitative evidence on how the health of care experienced children[1] (CEC) compares to other children in Scotland and in the UK. Invariably, the evidence that is available suggests that care experience is related to poorer health,[1–4] but often this is based on small sample sizes or without comparison to children who have not been in care.[2,3] The only population-wide evidence on the effects of care experience on health in Scotland (and the UK) comes from a linked administrative data study on dental health.[4] The study indicates considerable complexity in how different care experiences are related to health. Overall, CEC experience worse dental health outcomes compared to children who have not been in care, but there are significant differences in health outcomes by care type. For example, urgent and non-urgent dental needs were highest for those in home or kinship care and lower for children in foster care.

The cited research also shows that CEC are more likely to live in deprived areas compared to those who have not been in care, with half of all children in home and kinship care living in the most deprived areas. Importantly, differences in health outcomes between children persist after accounting for area deprivation.[4] Many previous studies have often not been able to account for area deprivation and family socioeconomic status (SES),[1] something that has a substantial effect on both health outcomes[5] and the chances of experiencing social care.[6]

In the UK, researchers have reported higher mortality,[7] poorer self-rated general and mental health,[8] and higher rates of some physical illnesses (epilepsy, cystic fibrosis, and cerebral palsy)[9] among CEC. There is also evidence of higher pregnancy rates among young women in care.[10] Research in Sweden, Finland and Canada underline high rates of mental health related problems like suicide and suicide attempts, psychiatric disorders and substance abuse among the care experienced population.[11–15] Other international research has shown evidence of higher rates of avoidable deaths (e.g. from causes that could have been prevented by timely medical care, or homicide and unintentional injuries)[16] and emergency department visits[17] among children in foster care.

Beyond this, there is very limited evidence on which objective health outcomes are most likely to differ between children who have and who have not experienced care and for this reason our study includes a wide variety of health measures, including hospitalisations, clinic attendances, prescriptions and mortality. Based on these findings and our discussions with the Centre for Excellence for Children's Care and Protection (CELCIS) and the Scottish Government, we also decided to include data on pregnancies for the young women in our cohort.

Administrative data linkage is the only feasible way of comparing a wide range of objective health outcomes between CEC and children in the general population (CGP) nationally, and in a representative way. Very few available data sets in Scotland and in the UK include details of both out of home or formal care and indicators of health. National surveys of children's health (e.g. Health Behaviour in School-aged Children study) and Scotland's Census include indicators of out of home and residential care, but these sources do not include any information on care history, or placement types and lengths. In Scotland, a child can also be "looked after" while living at home with their parent(s) under a home supervision order. For survey data, this means that it is not always possible

[1] 'Looked after children' is the statutory term for children in the care of local authorities, but often this excludes children who have left care. The term 'care experienced children' is now widely used to describe any person who has experience of being in care, regardless of their placement length, type or age.



For peer review only - http://bmjopen.bmj.com/site/about/guidelines.xhtml

to distinguish whether the child is formally considered "looked after" while living at home or with relatives. Finally, some surveys have too few CEC to analyse health outcomes for this subpopulation.

The lack of quantified evidence on the health of CEC has long been recognized as a major obstacle to evidence based policy making in the field.[18] The Children's Health in Care in Scotland (CHiCS) study provides the first robust nationwide evidence, with longitudinal data on care histories for CEC and a wide range of health outcomes for both cohorts of children. Improved knowledge about the health outcomes of CEC, particularly in comparison to CGP, will assist with the allocation of services by identifying precise areas of heightened needs, and will inform future research seeking to understand health inequalities between CEC and CGP.

## Cohort description

The CHiCS cohorts follow a range of health outcomes for two groups of school-aged children in publicly funded schools in Scotland; CEC and CGP (i.e. not care experienced). Children in Scotland usually start primary school at age four and a half to five and a half years old. Secondary school begins at age 11 or 12 for a compulsory four years with the following two years being optional. All children in the 2009 Pupil Census (PC), taken on September 21$^{st}$, were included in the baseline cohorts. The CEC cohort was then defined as all those children who were also on the 2009/2010 Scottish Government's Children Looked After (CLAS) return, collected between August 1$^{st}$, 2009 and July 31$^{st}$, 2010. The care histories of CEC were followed up to July 31$^{st}$, 2016 longitudinally, linking seven CLAS returns. The CGP cohort was defined as children who were on the 2009 PC that have not experienced care, i.e. not appeared on any of the CLAS returns from the first collection date on April 1$^{st}$ 2007 to the most recent date included in our study on July 31$^{st}$ 2016. The latter comparison was made to all possible CLAS returns available at the time to ensure that none of the general population children were or became looked after during the study.

The two cohorts of children were linked to all other data sources using the Scottish Candidate Number (SCN) present on the PC. The linkage of CLAS records to health is only possible via the SCN, and CEC who were not in school in 2009/2010, or had an invalid or missing SCN could not be included in the cohort. The 2009/10 CLAS return was chosen as the baseline cohort as the completeness of SCN on the CLAS return became high enough to make linkage between the CLAS return, the PC and health data representative. Details of SCN completeness for a single CLAS return has been published previously[19] and for our study this was improved further by the longitudinal linkage of multiple CLAS returns. The PC itself has nearly 100% coverage of all children in publicly funded schools, is frequently used in research and in provision of Accredited National Statistics.

Health outcomes for the CHiCS cohorts will be examined over the 7-year period between 2009 and 2016. The health data include the number of and diagnoses relating to hospital admissions (from Scottish Morbidity Records; SMR01/02/04) or speciality for outpatient clinic attendance (SMR00), number of and British National Formulary (BNF) chapter and section code for prescriptions (PIS), and cause of death (Supplementary Table S.1). For Accidents & Emergency (A&E) data only the number of attendances per year for each child was available. All the SMR records, PIS and A&E data are collected and shared by Public Health Scotland. The Birth and Death Registrations data are available from the National Records of Scotland. All included health, birth and death data have been widely used in research, undergo regular data quality checks and are considered high quality [20–22].

For hospitalisations, we also have access to outcomes from 1990 to account for past health. Previous research has identified differences in health service use (in primary care) among both mothers and



For peer review only - http://bmjopen.bmj.com/site/about/guidelines.xhtml

CEC before the child entered care.[23] For A&E data past events are recorded from 2007 but the individual level prescribing data is only available from 2009 [20]. For SMR02 and SMR04 admission reason was also recorded and will be used in research if the quality permits.

For CEC we have data on all care placements during the study period, including the start and end month and year of the placements (giving the length in months) and the care placement type. The majority of children (58%) had one care placement during the study, 18.5% had two, 9.5% three and 5.4% four placements, leaving 8.4% with five or more placements during the 7-year period. Table 1 shows that the most common placement types were at home under a Supervision Requirement (regular contact with social services) (31.2%), in foster care (total 28.8%) or with friends/relatives (17.7%). The mean placement length is 19 months (just over 1.5 years), but this varies considerably across placement types, with residential types generally having shorter placement lengths compared to living within a private (family) household (Table 1). The data also include an indicator if the child was in care before the study start date and the length and type of these placements. However, the quality of the earlier records is inconsistent and will require more analysis before we can determine if and how this can be used in research.

The individual level linkage process of the 10 data sources is described in Supplement Figure S.1 and follows the steps outlined previously for research on dental health.[19] Data cleaning steps and the size of the two cohorts are described in Figure 1. The key data sets in deriving the cohorts were the CLAS return and the PC. Twins and triplets were removed using information from Birth Registrations (the linkage is based on postcode, date of birth and sex, thus twins living at the same address are difficult to reliably link).

In total, 663,602 school-aged children were included in the study. Children were aged four to 17 years old at the start of the study and 11 to 24 years old by the end of follow-up in 2016. In total 13,831 (2.1%) were identified as CEC and 649,771 (97.9%) as CGP. There was a higher proportion of male CEC than male CGP (54.9% compared to 50.8%; see Table 2). On average, CEC tended to be older than CGP.  CEC were aged 11 years and 3 months on average (mean age in months=135, SD=42.2) and CGP aged 10 years and 10 months on average (mean age in months=130, SD=43.0), with a higher proportion of children in the CEC cohort born in the early 1990's. Differences in the age and sex distributions for all CEC and GCP with birth records is comparable to the differences in the age and sex distributions for all CEC and CGP. This suggests that children with birth records available are representative of all children in the study.

**Individual level socioeconomic profile**

In total, 88.6% and 88.0% of CEC and GCP respectively had birth records available with information including mothers' age at the time of birth and parental employment status (see Table 2, columns on data for children with birth records). On average, mothers of CEC were younger at birth (24.5 years old compared to 28.3 years). In total 56.1% of mothers of CEC were aged under 25 years old, compared to 26.1 % of mothers of CGP.

For children born before 1996, only one parent's occupation was recorded at birth (father's occupation if married, otherwise mother's occupation) and we therefore report parental employment status for children born before 1996. Parents of CEC were less likely to be in employment than parents of CGP (71.2% in employment compared to 92.8%). From 1996 onwards, both mother's and father's occupation were recorded for all births registered by married couples or for births that were jointly registered by unmarried couples, so we report both mother's and father's



For peer review only - http://bmjopen.bmj.com/site/about/guidelines.xhtml

employment status. Only 40% of mothers of CEC, born after 1995, were in employment compared to 78% of mothers of CGP. Fathers of CEC were also less likely to be in employment than fathers of CGP. Note also that the occupational status of CEC fathers was more likely to be missing (23.2% compared to 5.7%) indicating more absence amongst CEC fathers.

**Area level socioeconomic profile**

Birth records include information on area of residence at birth (2001 data zone) and the PC records information on area of residence (2001 data zone) at the start of the study in 2009. We linked data zones at birth to the 2004 Scottish Index of Multiple Deprivation (SIMD) and linked data zones at the start of the study in 2009 to SIMD 2009v2. Table 3 gives the SIMD quintiles for area of birth for all children with birth records, for area of residence at start of study in 2009 for all children with birth records, and for area of residence at start of study in 2009 for all children in the study. In total, 59% of CEC (with birth records) lived in the most deprived quintile of deprivation compared to 25% of CGP at birth. By 2009, 41% of CEC (with birth records) were living in the most deprived quintile, perhaps reflecting residential moves into a less deprived area following birth and being taken into care.

The data zones can also be linked to Health Boards and to the Scottish Government Urban Rural Classification 2003/04 (at birth) and 2009/10 (in 2009), used to classify small areas as urban, rural or remote (Supplementary Table S.2). Scotland is divided into 14 Health Boards which have responsibility for health protection, promotion, and the delivery of services to their population. Social care is the responsibility of local government through Scotland's 32 local authorities. In terms of the urban-rural classification there was a shift for CEC away from large urban areas between birth and 2009 (50% in large urban areas at birth compared to 40% in 2009 for CEC with birth records). Perhaps unsurprisingly, CEC were also more likely to have changed data zones between birth and the start of the study in 2009 (Supplementary Table S.3). Only 12.4% of CEC were living in the same data zone of residence at birth by the beginning of the study. CEC were also more likely to have missing information on data zone of residence; highlighting one of the difficulties in studying hard-to-reach populations. In total 10% of CEC did not have residential details recorded in the Pupil Census, compared to just 0.04% in the general population.

**National comparisons**

Our cohort of CEC include those who were on the 2009/2010 CLAS return and in the 2009 PC. To give an estimate how well we capture the whole population of CEC and CGP in Scotland, we compare all children in our CEC cohort to Scottish Government National Children's Social Work Statistics (Supplementary Table S.4). Compared to national statistics on children in care, we see a very similar sex distribution with around 55% males and 45% females in care. Our age distribution varies in the youngest age group (0-4 years) as we include only school-aged children in our cohort and therefore only have a small proportion of four-year olds who were registered for school. For ages 5-15 we are capturing very similar numbers of CEC compared to the published national statistics.

We also compared all children in our study (CEC and CGP; n=663,602) to Scotland's 2011 census population aged 0-19 years old (Supplementary Table S.5). The comparison to the 2011 population census shows we have captured a high proportion of children of compulsory school age (95.5% of 5–11-year-olds and 93.4% of 12-15-year-olds). Note that for ages 5-15 we are including about 95% of



For peer review only - http://bmjopen.bmj.com/site/about/guidelines.xhtml

the population in Scotland. We also have captured 98.1% of the 2009 Pupil Census population of 676,740 pupils.[24] In conclusion, these comparisons suggest that our two cohorts are capturing a very high proportion of all school-aged children in Scotland and our study is representative of this population.

## Patient and Public Involvement Statement

We collaborated with CELCIS when planning and designing this research project. We have set up an Advisory Group including representatives from children's charities and public authorities responsible for the welfare of children and CEC to help guide and contextualize the research, and undertake knowledge exchange and user engagement programme. The planned knowledge exchange and user engagement programme will act as patient and public involvement and will be undertaken as the research progresses in 2021/2022.

## Findings to date

The descriptive analysis of the data in Table 4 shows that the proportion of CEC who have had at least one event recorded in any of the health data sources is higher compared to CGP for all data sets except for prescriptions. The biggest differences are evident for deaths, with CEC have 5.5 times higher mortality compared to CGP ($CEC_{rate}$ = 83.5, $CGP_{rate}$ = 15.3), and hospitalisations for maternity (CEC have 4.3 times higher rates in SMR02; $CEC_{rate}$ = 158.5, $CGP_{rate}$ = 36.6) and mental health inpatient and day cases (CEC have 5.2 times higher rates in SMR04; $CEC_{rate}$ = 923.2, $CGP_{rate}$ = 179.2). CEC also experience substantially more health events (hospital visits, prescriptions) per child in all health data sets, with the biggest differences evident for SMR02, SMR04 and A&E attendances. A similar proportion of CEC and CGP have received at least one prescription during the study period, but the average number of prescriptions is higher for CEC, meaning that, on average, CEC received more prescriptions.

The next tables give the most common prescriptions (Table 5), outpatient clinic specialties (Table 6), and diagnosed condition for acute inpatient admissions (Table 7). (For coding schemas see Supplementary Table S.5.) The outcomes are ordered from largest to smallest by the total number of events for each health outcome and separately for the two cohorts. The resulting different ordering for the two cohorts is intentional and aims to highlight the very marked differences in health between the two cohorts. The tables clearly show that the reasons for contacting the health services are dissimilar between the two cohorts. For example, a higher proportion of the CEC cohort have had prescriptions for depression (CEC = 19.7%, CGP = 8.1%), psychiatric outpatient clinic attendances (CEC = 20.1%, CGP = 5.5%) and acute inpatient admissions due to mental and behavioural disorders (CEC = 2.2%, CGP = 0.3%). The proportion of CEC hospitalised due to injuries, drug poisoning and other external causes is also higher compared to CGP (for drug poisoning CEC = 6.1%, CGP = 0.9%). The differences between the two cohorts in age-standardised rates for these prescriptions and hospitalisations are similarly notable.

A higher proportion of care experienced young women have had outpatient obstetrics (CEC = 17.7%, CGP = 3.8%), gynaecology (CEC = 14.5%, CGP = 8.0%) and midwifery (CEC = 4.1%, CGP = 0.4%) clinic attendances, and have had an abortion (CEC = 7.1%, CGP = 3.8%). The age-standardised rates for these hospital attendances are also higher for the care experienced young women compared to the women in the general population. The proportion of care experienced young women who have been



For peer review only - http://bmjopen.bmj.com/site/about/guidelines.xhtml

prescribed contraceptives is similar to that of young women in the general population (CEC = 42.5%, CGP = 41.7%), however the total number of contraceptive prescriptions and the age-standardized rates are higher for the women in the general population ($CEC_{rate}$ = 218.4, $CGP_{rate}$ = 349.0). Without additional data on the type of contraceptive prescribed (long-acting, e.g. implants, *versus* not long-acting, e.g. oral contraceptives) we cannot say what drives this difference. However, previous research suggests that women from more deprived areas are more likely to received long-acting reversible contraceptives,[25] and this could explain why the care experienced women in our cohort have fewer prescriptions compared to the women in the general population.

These initial results show substantial differences in health and health service use between the two cohorts. First, the proportion of children who have been in contact with the health services is higher for the CEC compared to the CGP. Second, CEC have more frequent contact with health services across all data sets. Third, many of the reasons for contacting health services are different for the two cohorts, with the CEC having more frequent contact related to mental, sexual and reproductive health. The level of contact with the health services for some causes (e.g. outpatient attendance for ear, nose and throat diseases) is similar for the two cohorts.

The CHiCS cohorts outlined here show substantial differences in the socioeconomic background and health between children who have experienced care and those who have not. They provide a unique opportunity for the first population-wide evidence on objective health outcomes for an understudied and vulnerable population of children. Next, our research will focus on explaining the differences in the health outcomes between the two cohorts of children using event history analysis on matched cohorts.

## Strengths and limitations

The CHiCS project is the first national longitudinal data collection in the UK that compares the health of CEC to CGP. In addition to the large population-wide sample and a 7-year follow-up, other main strengths of the study include the wide range of health outcomes available, along with, the high quality and representativeness of the data. Together these will allow for robust and detailed results on a very vulnerable population group.

The weaknesses of the study include errors in the individual data sets and in data linkage. Of the health data sets, the SMR00 (outpatient attendances) is of weakest quality with more inconsistent or inaccurate recording of sex. However, the noted errors in recording data and in linkage only affect approximately 3% of children in both cohorts and for the rest of the children we did not note any inconsistencies (e.g. in sex or age) across the data sets.

Another weakness is that the PC excludes children educated at home or at independent schools. However, only about 25-30,000 children are educated in independent schools (4% of all children attending school in Scotland).[26] Based on this and on the comparison of our data to the population census estimates of the number of children in Scotland (supplement Table S.5), we are confident that our data are representative of school children in Scotland.

Additional potential confounders on parents' background over and above what has been presented (i.e. mother's age at birth, parental employment status at birth, area of residence at birth) were not available for this project. However, future research may be able to link maternal health to these data through Birth Registrations.



For peer review only - http://bmjopen.bmj.com/site/about/guidelines.xhtml

As this study has highlighted mental, sexual and reproductive health as areas with biggest health differences between the two cohorts, future research would benefit from more focused attention and detailed data collection in a specific area of health. This could include more detailed data on prescriptions (e.g. to distinguish long-acting and other contraceptives) and, where possible, linking in smaller less often used community health or primary care data sets.

## Collaboration

To access the data, the authors applied for data access to the Public Benefit and Privacy Panel for Health and Social Care and to the Scottish Government's Statistics Public Benefit and Privacy Panel. After data access was approved by both organizations a data sharing agreement between the University of Glasgow and the Scottish Government was agreed. Due to the sensitivity of the data, it will not be made publicly available.

We encourage collaboration from other researchers, charities and public bodies interested in this research that fit with the original aims of this study. We also encourage collaborations to extend the follow-up period of both cohorts to 2020 and beyond to study the effects of national lock-downs on the health of young adults and care leavers.

## Ethics

Ethical approval was obtained from the University of Glasgow College of Medicine, Veterinary and Life Sciences Ethics Committee [Project No: 200160031].

## Funding

This work was supported by the Economic and Social Research Council [grant number ES/T000120/1]. The data linkage and access to the National Safe Haven was facilitated by the Urban Big Data Centre [grant number ES/L011921/1]. MA, DB and AHL are also funded by the Medical Research Council (MC_UU_00022/2) and the Scottish Government Chief Scientist Office (SPHSU17).

## Acknowledgements

The authors would like to acknowledge the support of the eDRIS Team (Public Health Scotland) for their involvement in obtaining approvals, provisioning and linking data and the use of the secure analytical platform within the National Safe Haven. The National Records of Scotland undertook the indexing and provided the linkage key for this study. The authors would also like to acknowledge the Scottish Exchange of Data (ScotXed) for allowing us to access their data and the CHiCS project Advisory Group members for their engagement and feedback on the initial study results.

## Author contributions

MA and DB conceived the research idea and carried out most of the research and writing. CTBL and CM assisted in initial data analysis and provided feedback on the draft of this paper. AL and MH



For peer review only - http://bmjopen.bmj.com/site/about/guidelines.xhtml

contributed to the research design and have provided important feedback throughout the project and writing of this paper.

## Competing interest

No competing interests declared.

## Data availability statement

These data can be accessed through applications to the Public Benefit and Privacy Panel for Health and Social Care (https://www.informationgovernance.scot.nhs.uk/pbpphsc/) and to the Scottish Government's Statistics Public Benefit and Privacy Panel (https://www.gov.scot/publications/scottish-government-statistics-request-our-data/).

For peer review only



For peer review only - http://bmjopen.bmj.com/site/about/guidelines.xhtml

## References


1. Meltzer H, Lader D, Corbin T, Goodman R, Ford T. The mental health of young people looked after by local authorities in Scotland. Office for National Statistics; 2004.

2. Ridley J, McCluskey S. Exploring the Perceptions of Young People in Care and Care Leavers of the Health needs. Scott J Resid Care. 2003;2(1):55–65.

3. Reilly C, Johnson DR, Ferguson K. Validation of the Massachusetts Youth Screening Instrument with a looked after population. Clin Child Psychol Psychiatry. 2018 Sep;1359104518799119.

4. McMahon AD, Elliott L, Macpherson LM, Sharpe KH, Connelly G, Milligan I, et al. Inequalities in the dental health needs and access to dental services among looked after children in Scotland: a population data linkage study. Arch Dis Child [Internet]. 2017; Available from: https://adc.bmj.com/content/early/2017/08/18/archdischild-2016-312389

5. Brown D, Allik M, Dundas R, Leyland AH. All-cause and cause-specific mortality in Scotland 1981-2011 by age, sex and deprivation: a population-based study. Eur J Public Health [Internet]. 2019 Feb; Available from: https://dx.doi.org/10.1093/eurpub/ckz010

6. Bywaters P, Scourfield J, Jones C, Sparks T, Elliott M, Hooper J, et al. Child welfare inequalities in the four nations of the UK. J Soc Work. 2018 Sep;1468017318793479.

7. Murray ET, Lacey R, Maughan B, Sacker A. Association of childhood out-of-home care status with all-cause mortality up to 42-years later: Office of National Statistics Longitudinal Study. BMC Public Health. 2020;20(1):735.

8. Viner RM, Taylor B. Adult health and social outcomes of children who have been in public care: population-based study. Pediatrics. 2005 Apr;115(4):894–9.

9. Martin A, Ford T, Goodman R, Meltzer H, Logan S. Physical illness in looked-after children: a cross-sectional study. Arch Dis Child. 2014;99(2):103–7.

10. Craine N, Midgley C, Zou L, Evans H, Whitaker R, Lyons M. Elevated teenage conception risk amongst looked after children; a national audit. Public Health. 2014 Jul;128(7):668–70.

11. Berlin M, Vinnerljung B, Hjern A. School performance in primary school and psychosocial problems in young adulthood among care leavers from long term foster care. Child Youth Serv Rev. 2011 Dec;33(12):2489–97.

12. Taussig HN, Harpin SB, Maguire SA. Suicidality among preadolescent maltreated children in foster care. Child Maltreat. 2014/02/24 ed. 2014 Feb;19(1):17–26.

13. Vinnerljung B, Hjern A, Lindblad F. Suicide attempts and severe psychiatric morbidity among former child welfare clients - a national cohort study. J Child Psychol Psychiatry. 2006 Jul;47(7):723–33.

14. Vinnerljung B, Ribe M. Mortality after care among young adult foster children in Sweden. Int J Soc Welf. 2001;10(3):164–73.




For peer review only - http://bmjopen.bmj.com/site/about/guidelines.xhtml

15. Katz LY, Au W, Singal D, Brownell M, Roos N, Martens PJ, et al. Suicide and suicide attempts in children and adolescents in the child welfare system. CMAJ Can Med Assoc J J Assoc Medicale Can. 2011 Nov;183(17):1977–81.

16. Hjern A, Vinnerljung B, Lindblad F. Avoidable mortality among child welfare recipients and intercountry adoptees: a national cohort study. J Epidemiol Community Health. 2004;58(5):412–7.

17. Rubin DM, Alessandrini EA, Feudtner C, Localio AR, Hadley T. Placement Changes and Emergency Department Visits in the First Year of Foster Care. Pediatrics. 2004 Sep;114(3):e354.

18. Scott S, Hattie R, Tannahill C. Looked After Children in Glasgow and Scotland: A Health Needs Assessment. ScotPHN; 2013.

19. Clark D, King A, Sharpe K, Connelly G, Elliott L, Macpherson LMD, et al. Linking routinely collected social work, education and health data to enable monitoring of the health and health care of school-aged children in state care ('looked after children') in Scotland: a national demonstration project. Public Health. 2017 Sep;150:101–11.

20. Alvarez-Madrazo S, McTaggart S, Nangle C, Nicholson E, Bennie M. Data Resource Profile: The Scottish National Prescribing Information System (PIS). Int J Epidemiol. 2016/05/10 ed. 2016 Jun;45(3):714–715f.

21. PHS PHS. About Data Quality Assurance [Internet]. 2021. Available from: https://www.isdscotland.org/Products-and-Services/Data-Quality/About-Data-Quality-Assurance.asp

22. NRS NR of S. Quality assurance of administrative data used in population statistics: Births and deaths data [Internet]. 2020 Oct. Available from: https://www.nrscotland.gov.uk/files/statistics/population/quality-assurance/qaad-births-deaths-data.pdf

23. Simkiss DE, Spencer NJ, Stallard N, Thorogood M. Health service use in families where children enter public care: a nested case control study using the General Practice Research Database. BMC Health Serv Res. 2012;12(1):65.

24. The Scottish Government. Pupil census: supplementary statistics [Internet]. 2021 [cited 2021 Jun 17]. Available from: https://www.gov.scot/publications/pupil-census-supplementary-statistics/

25. Morgan CR, Liu H. The relationship between area deprivation and prescription of long-acting reversible contraception in women of reproductive age in Lothian, Scotland, UK. BMJ Sex Reprod Health. 2017;43(4):281–8.

26. SCIS SC of IS. Facts and Figures [Internet]. 2021 [cited 2021 Jun 16]. Available from: https://www.scis.org.uk/facts-and-figures/

For peer review only

# Tables

## *Table 1: Number, percent and length of placements during study period by the type of placement*

| Placement type | Placements | | Length (months) | |
|---|---|---|---|---|
| | N | % | Mean | Median |
| Private household placement | | | | |
| At home | 8,716 | 31.2 | 16.7 | 12 |
| With foster carers provided by LA | 5,705 | 20.4 | 23.6 | 13 |
| With foster carers purchased by LA | 2,362 | 8.4 | 25.4 | 15 |
| With friends/relatives | 4,958 | 17.7 | 25.6 | 17 |
| In other community | 349 | 1.2 | 6.4 | 4 |
| With prospective adopters | 180 | 0.6 | 15.2 | 12 |
| Residential placements | | | | |
| In LA home | 2,545 | 9.1 | 11.2 | 6 |
| In residential school | 1,051 | 3.8 | 16.3 | 12 |
| In secure accommodation | 729 | 2.6 | 4.8 | 3 |
| In voluntary home | 352 | 1.3 | 13.9 | 8 |
| In crisis care | 85 | 0.3 | 6.0 | 2 |
| Other residential | 929 | 3.3 | 10.9 | 5 |
| Total | 27,961 | 100 | 19.2 | 11 |

Note: LA – Local Authority

## *Table 2: Individual level socioeconomic profiles of CEC and CGP cohorts*

| | **Children with birth records** | | | | **All children** | | | |
|---|---|---|---|---|---|---|---|---|
| | **CGP** | | **CEC** | | **CGP** | | **CEC** | |
| | **N** | **%** | **N** | **%** | **N** | **%** | **N** | **%** |
| **Number (%) of children** | **571,702** | **88.0** | **12,250** | **88.6** | **649,771** | **100.0** | **13,831** | **100.0** |
| **Female** | 281,221 | 49.2 | 5,565 | 45.4 | 319,438 | 49.2 | 6,274 | 45.4 |
| **Male** | 290,481 | 50.8 | 6,685 | 54.6 | 330,333 | 50.8 | 7,557 | 54.6 |
| **Age in months** | | | | | | | | |
| *mean (SD)* | *130 (43.0)* | | *135 (42.2)* | | *131 (43.0)* | | *136 (42.3)* | |
| **Year of birth** | | | | | | | | |
| 1990-1995 | 160,644 | 28.1 | 4,114 | 33.6 | 184,923 | 28.5 | 4,830 | 34.9 |
| 1996-2000 | 234,120 | 41.0 | 4,862 | 39.7 | 265,453 | 40.9 | 5,413 | 39.1 |
| 2001-2004 | 176,938 | 30.9 | 3,274 | 26.7 | 199,395 | 30.7 | 3,588 | 25.9 |
| **For children with birth records:** | | | | | | | | |
| **Mother's age at birth** | | | | | | | | |
| *mean (SD)* | *28.3 (5.7)* | | *24.5 (5.8)* | | | | | |
| Aged under 20 | 43,624 | 7.6 | 2,792 | 22.8 | | | | |
| Aged 20-24 | 105,653 | 18.5 | 4,066 | 33.2 | | | | |
| Aged 25-29 | 175,114 | 30.6 | 2,898 | 23.7 | | | | |
| Aged 30-34 | 167,634 | 29.3 | 1,664 | 13.6 | | | | |
| Aged 35-39 | 68,442 | 12.0 | 682 | 5.6 | | | | |
| Aged 40+ | 10,737 | 1.9 | 130 | 1.1 | | | | |
| *Missing* | *498* | *0.1* | *18* | *0.1* | | | | |
| **Parental employment status at birth** | | | | | | | | |



For peer review only - http://bmjopen.bmj.com/site/about/guidelines.xhtml

| | | | | |
|---|---|---|---|---|
| *Parental (for births 1990-1995)* | | | | |
| Employee | 117,526 | 73.2 | 2,715 | 66.0 |
| Manager | 14,836 | 9.2 | 84 | 2.0 |
| Supervisor | 4,788 | 3.0 | 35 | 0.9 |
| Self-employed (with employees) | 4,919 | 3.1 | 27 | 0.7 |
| Self-employed (without employees) | 6,955 | 4.3 | 67 | 1.6 |
| Student/Unemployed/Not available | 11,620 | 7.2 | 1,186 | 28.8 |
| *Missing* | *0* | *0.0* | *0* | *0.0* |
| *Mother (for births 1996-2004)* | | | | |
| Employee | 271,711 | 66.1 | 3,138 | 38.6 |
| Manager | 28,201 | 6.9 | 58 | 0.7 |
| Supervisor | 9,315 | 2.3 | 39 | 0.5 |
| Self-employed (with employees) | 3,811 | 0.9 | 7 | 0.1 |
| Self-employed (without employees) | 7,558 | 1.8 | 33 | 0.4 |
| Student/Unemployed/Not available | 90,425 | 22.0 | 4,861 | 59.7 |
| *Missing* | *37* | 0.0 | *0* | 0.0 |
| *Father (for births 1996-2004)* | | | | |
| Employee | 274,748 | 66.8 | 4,467 | 54.9 |
| Manager | 44,524 | 10.8 | 130 | 1.6 |
| Supervisor | 12,791 | 3.1 | 61 | 0.7 |
| Self-employed (with employees) | 15,036 | 3.7 | 79 | 1.0 |
| Self-employed (without employees) | 24,631 | 6.0 | 285 | 3.5 |
| Student/Unemployed/Not available | 15,889 | 3.9 | 1,227 | 15.1 |
| *Missing* | *23,439* | *5.7* | *1,887* | *23.2* |

*Table 3: Deprivation level of area of residence of CEC and CGP cohorts*

| | **All children with birth records** | | | | **All children with birth records** | | | | **All children** | | | |
|---|---|---|---|---|---|---|---|---|---|---|---|---|
| | ***Area of residence at birth*** | | | | ***Area of residence in 2009*** | | | | ***Area of residence in 2009*** | | | |
| | **CGP** | | **CEC** | | **CGP** | | **CEC** | | **CGP** | | **CEC** | |
| | **N** | **%** | **N** | **%** | **N** | **%** | **N** | **%** | **N** | **%** | **N** | **%** |
| **Number (%) of children** | **571,702** | **88.0** | **12,250** | **88.6** | **571,702** | **88.0** | **12,250** | **88.6** | **649,771** | **100.0** | **13,831** | **100.0** |
| **Quintile of deprivation** | | | | | | | | | | | | |
| Q1 (most deprived) | 142,253 | 24.9 | 7,166 | 58.5 | 123,331 | 21.6 | 5,061 | 41.3 | 140,120 | 21.6 | 5,591 | 40.4 |
| Q2 | 116,146 | 20.3 | 2,650 | 21.6 | 112,764 | 19.7 | 2,636 | 21.5 | 125,764 | 19.4 | 2,914 | 21.1 |
| Q3 | 107,180 | 18.7 | 1,420 | 11.6 | 110,157 | 19.3 | 1,641 | 13.4 | 125,704 | 19.3 | 1,852 | 13.4 |
| Q4 | 103,610 | 18.1 | 694 | 5.7 | 112,379 | 19.7 | 1,116 | 9.1 | 129,566 | 19.9 | 1,268 | 9.2 |
| Q5 (least deprived) | 101,665 | 17.8 | 303 | 2.5 | 112,846 | 19.7 | 613 | 5.0 | 127,979 | 19.7 | 696 | 5.0 |
| *Missing* | *858* | *0.2* | *17* | *0.1* | *225* | *0.0* | *1,183* | *9.7* | *638* | 0.1 | *1,510* | 10.9 |



For peer review only - http://bmjopen.bmj.com/site/about/guidelines.xhtml

*Table 4: Main health outcomes for the two cohorts, 2009-2016*

| | Children with at least one event | | | | Mean per child | | Rate* | | Ratio of rates |
|---|---|---|---|---|---|---|---|---|---|
| | CGP | | CEC | | CGP | CEC | CGP | CEC | CEC:CGP |
| | N | %** | N | % | | | | | |
| **Total children** | 649,771 | 100 | 13,831 | 100 | | | | | |
| **Total female** | 319,438 | 49.2 | 6,274 | 45.4 | | | | | |
| Deaths | 746 | 0.1 | 78 | 0.6 | 0.11 | 0.56 | 15.3 | 83.5 | 5.48 |
| PIS | 603,628 | 92.9 | 12,597 | 91.1 | 28.32 | 34.71 | 3642.3 | 4446.0 | 1.22 |
| SMR00 | 382,590 | 58.9 | 9,427 | 68.2 | 6.30 | 8.09 | 469.7 | 736.1 | 1.57 |
| SMR01 | 179,551 | 27.6 | 5,404 | 39.1 | 2.22 | 2.60 | 94.0 | 150.5 | 1.60 |
| SMR02*** | 14,269 | 4.5 | 1,302 | 20.8 | 2.82 | 3.24 | 36.6 | 158.5 | 4.33 |
| SMR04 | 2,197 | 0.3 | 323 | 2.3 | 3.56 | 2.90 | 179.2 | 923.2 | 5.15 |
| A&E | 434,528 | 66.9 | 10,826 | 78.3 | 3.36 | 5.86 | 273.2 | 572.1 | 2.09 |

Note: SMR00 - Outpatient Attendance; SMR01 - General/ Acute Inpatient and Day Case; SMR02 - Maternity Inpatient and Day Case; SMR04 - Mental Health Inpatient and Day Case
*Age standardized for ages 0-24 using the 2013 European standard population per 1000 person-years (PY). For deaths and SMR04, rates are shown per 100,000 PY.
**Percent from cohort.
***Calculated for female members of the cohort.

For peer review only



For peer review only - http://bmjopen.bmj.com/site/about/guidelines.xhtml

*Table 5: PIS – Prescribing Information System*

| | CGP | | | | | | CEC | | | | |
|---|---|---|---|---|---|---|---|---|---|---|---|
| **BNF Chapter/Section** | **Prescriptions** | | **Children** | | | **BNF Chapter/Section** | **Prescriptions** | | **Children** | | |
| | **N** | **%** | **N** | **%** | **Rate*** | | **N** | **%** | **N** | **%** | **Rate*** |
| 3 - Respiratory system | 3,350,333 | 19.6 | 334,231 | 51.4 | 689.1 | 4 - Central Nervous System | 116,988 | 26.8 | 9,215 | 66.6 | 1265.2 |
| 301 - Bronchodilators | 1,164,625 | 6.8 | 130,986 | 20.2 | 241.5 | 407 - Analgesics | 31,394 | 7.2 | 7,570 | 54.7 | 393.4 |
| 304 - Antihistamines | 1,108,966 | 6.5 | 236,979 | 36.5 | 215.6 | 404 - CNS and ADHD | 28,455 | 6.5 | 794 | 5.7 | 224.3 |
| 302 - Corticosteroids | 750,694 | 4.4 | 76,544 | 11.8 | 157.1 | 403 - Antidepressant | 20,922 | 4.8 | 2,723 | 19.7 | 260.2 |
| 13 - Skin | 3,260,944 | 19.1 | 456,524 | 70.3 | 690.9 | 3 - Respiratory system | 71,972 | 16.5 | 7,537 | 54.5 | 713.4 |
| | | | | | | 301 - Bronchodilators | 27,163 | 6.2 | 2,897 | 20.9 | 265.5 |
| 4 - Central Nervous System | 2,301,960 | 13.5 | 327,896 | 50.5 | 530.5 | 302 - Corticosteroids | 16,881 | 3.9 | 1,625 | 11.7 | 169.9 |
| 407 - Analgesics | 1,020,520 | 6.0 | 279,953 | 43.1 | 245.1 | 304 - Antihistamines | 18,663 | 4.3 | 4,779 | 34.6 | 169.5 |
| 403 - Antidepressant | 398,369 | 2.33 | 52,768 | 8.1 | 114.4 | | | | | | |
| 404 - CNS and ADHD | 273,164 | 1.6 | 7548 | 1.2 | 41.16 | 13 - Skin | 62,960 | 14.4 | 9,707 | 70.2 | 621.5 |
| 5 - Infections | 2,234,038 | 13.1 | 470,671 | 72.4 | 487.8 | 5 - Infections | 45,127 | 10.3 | 9,520 | 68.8 | 448.8 |
| 501 - Antibacterial drugs | 2,055,439 | 12.0 | 454,724 | 70.0 | 444.7 | 501 - Antibacterial drugs | 41,721 | 9.5 | 9,178 | 66.4 | 411.0 |
| 703 - Contraceptives** | 779,348 | 4.6 | 133,343 | 41.7 | 349.0 | 1001 - Drugs used in rheumatic diseases & gout | 15,104 | 3.5 | 6,041 | 43.7 | 146.3 |
| 1001 - Drugs used in rheumatic diseases & gout | 638,973 | 3.7 | 258,205 | 39.7 | 132.6 | 601 - Diabetes drugs | 14,391 | 3.3 | 139 | 1.0 | 124.9 |
| 601 - Diabetes drugs | 592,741 | 3.5 | 5,396 | 0.8 | 105.5 | 703 - Contraceptives** | 11,552 | 2.6 | 2,685 | 42.8 | 218.4 |

Note: BNF sections are indented.
*Age standardized for age groups 0-24 using the 2013 European standard population per 1000 person-years.
**For contraceptives, the percent of children and the age-standardized rate are calculated for the female members of the cohort.

For peer review only

*Table 6: SMR00 – Outpatient Attendances*

| CGP | | | | | |
|---|---|---|---|---|---|
| **Clinic specialty** | **Attendances** | | **Children** | | |
| | **N** | **%** | **N** | **%** | **Rate*** |
| D5 - Orthodontics | 492,407 | 20.4 | 51,950 | 8.0 | 67.5 |
| C8 - Trauma & Orthopaedic Surgery | 318,094 | 13.2 | 121,722 | 18.7 | 58.8 |
| AF - Paediatrics | 233,131 | 9.7 | 66,860 | 10.3 | 49.3 |
| A7 - Dermatology | 203,359 | 8.4 | 61,886 | 9.5 | 40.3 |
| Psychiatry (all) | 195,907 | 8.1 | 35,802 | 5.5 | 32.6 |
| C5 - Ear, Nose & Throat | 133,917 | 5.6 | 63,502 | 9.8 | 32.2 |
| Dentistry (all) | 104,934 | 4.4 | 24,581 | 3.8 | 22.9 |
| C7 - Ophthalmology | 80,998 | 3.4 | 32,000 | 4.9 | 21.7 |
| A9 - Gastroenterology | 47,755 | 2.0 | 14,737 | 2.3 | 10.8 |
| Obstetrics** (all) | 47,015 | 2.0 | 12,135 | 3.8 | 26.2 |
| AH - Neurology | 46,675 | 1.9 | 17,394 | 2.7 | 9.4 |
| F2 – Gynaecology** | 45,324 | 1.9 | 25,508 | 8.0 | 23.6 |
| C9 - Plastic Surgery | 44,880 | 1.9 | 16,511 | 2.5 | 8.8 |
| T2 – Midwifery** | 20,510 | 0.9 | 2,561 | 0.4 | 11.3 |
| G5 - Learning Disability (Mental Handicap) | 5,033 | 0.2 | 1,026 | 0.2 | 1.4 |

| CEC | | | | | |
|---|---|---|---|---|---|
| **Clinic specialty** | **Attendances** | | **Children** | | |
| | **N** | **%** | **N** | **%** | **Rate*** |
| Psychiatry (all) | 20,170 | 26.5 | 2,781 | 20.1 | 163.2 |
| C8 - Trauma & Orthopaedic Surgery | 7,507 | 9.8 | 2,805 | 20.3 | 68.4 |
| AF - Paediatrics | 7,015 | 9.2 | 2,034 | 14.7 | 79.7 |
| D5 - Orthodontics | 6,180 | 8.1 | 851 | 6.2 | 42.2 |
| Obstetrics** (all) | 5,868 | 7.7 | 1,113 | 17.7 | 131.6 |
| C5 - Ear, Nose & Throat | 3,088 | 4.1 | 1,452 | 10.5 | 38.0 |
| Dentistry (all) | 2,957 | 3.9 | 797 | 5.8 | 30.8 |
| T2 – Midwifery** | 2,694 | 3.5 | 255 | 4.1 | 65.4 |
| A7 - Dermatology | 2,602 | 3.4 | 858 | 6.2 | 23.5 |
| G5 - Learning Disability (Mental Handicap) | 2,134 | 2.8 | 195 | 1.4 | 29.9 |
| C7 - Ophthalmology | 2,067 | 2.7 | 869 | 6.3 | 36.4 |
| F2 – Gynaecology** | 1,844 | 2.4 | 908 | 14.5 | 44.1 |
| AH - Neurology | 1,593 | 2.1 | 555 | 4.0 | 15.0 |
| C9 - Plastic Surgery | 1,070 | 1.4 | 393 | 2.8 | 11.0 |
| A9 - Gastroenterology | 725 | 1.0 | 273 | 2.0 | 6.8 |

*Age standardized for age groups 0-24 using the 2013 European standard population per 1000 person-years.

**For obstetrics, gynaecology and midwifery the percent of children and the age-standardised rate are calculated for the female members of the cohort.

For peer review only

*Table 7: SMR01 – General/Acute Inpatient and Day Cases by ICD-10 chapters*

**CGP**

| Main diagnosis | Attendances N | Attendances % | Children N | Children % | Rate* |
|---|---|---|---|---|---|
| Diseases of the digestive system | 61,704 | 15.5 | 41,483 | 6.4 | 17.0 |
|     Dental caries (K02) | 14,084 | 3.5 | 13,301 | 2.0 | 7.0 |
| Injury, poisoning and other consequences of external causes | 55,038 | 13.8 | 42,002 | 6.5 | 12.0 |
|     Other injury | 28,726 | 7.2 | 22,899 | 3.5 | 6.3 |
|     Head injury | 11,095 | 2.8 | 9,745 | 1.5 | 2.7 |
|     Drug poisoning | 9,251 | 2.3 | 6,129 | 0.9 | 1.8 |
|     Hand injury | 5,966 | 1.5 | 5,435 | 0.8 | 1.3 |
| Symptoms, signs and abnormal clinical and laboratory findings | 50,192 | 12.6 | 33,271 | 5.1 | 12.0 |
| Diseases of the respiratory system | 39,226 | 9.8 | 25,628 | 3.9 | 10.5 |
|     Asthma | 9,172 | 2.3 | 7,562 | 1.2 | 2.7 |
| Diseases of the genitourinary system | 25,776 | 6.5 | 16,600 | 2.6 | 5.4 |
| Diseases of the musculoskeletal system and connective tissue | 23,431 | 5.9 | 12,365 | 1.9 | 4.5 |
| Neoplasms | 21,800 | 5.5 | 5,340 | 0.8 | 4.9 |
| Examination, observation, etc | 19,259 | 4.8 | 12,918 | 2.0 | 4.4 |
| Obstetric** | 15,099 | 3.8 | 13,089 | 4.1 | 6.9 |
|     Medical abortion (O04)** | 13,574 | 3.4 | 12,107 | 3.8 | 6.1 |
| Endocrine, nutritional and metabolic diseases | 13,910 | 3.5 | 3,978 | 0.6 | 2.6 |
| Mental and behavioural disorders | 2,646 | 0.664 | 2,207 | 0.3 | 0.5 |

**CEC**

| Main diagnosis | Attendances N | Attendances % | Children N | Children % | Rate* |
|---|---|---|---|---|---|
| Injury, poisoning and other consequences of external causes | 3,900 | 27.7 | 2,198 | 15.9 | 36.2 |
|     Drug poisoning | 1,671 | 11.9 | 849 | 6.1 | 13.0 |
|     Other injury | 1,262 | 9.0 | 895 | 6.5 | 10.7 |
|     Head injury | 626 | 4.5 | 514 | 3.7 | 5.2 |
|     Hand Injury | 341 | 2.4 | 298 | 2.2 | 2.9 |
| Symptoms, signs and abnormal clinical and laboratory findings | 1,811 | 12.9 | 1,059 | 7.7 | 17.3 |
| Diseases of the digestive system | 1,626 | 11.6 | 1,158 | 8.4 | 27.2 |
|     Dental caries (K02) | 631 | 4.5 | 590 | 4.3 | 17.7 |
| Diseases of the respiratory system | 1,108 | 7.9 | 618 | 4.5 | 12.4 |
|     Asthma | 317 | 2.3 | 116 | 0.8 | 2.9 |
| Diseases of the genitourinary system | 867 | 6.2 | 463 | 3.3 | 7.3 |
| Obstetric** | 702 | 5.0 | 539 | 8.6 | 15.5 |
|     Medical abortion (O04)** | 538 | 3.8 | 447 | 7.1 | 11.3 |
| Endocrine, nutritional and metabolic diseases | 604 | 4.3 | 114 | 0.8 | 5.7 |
| Examination, observation, etc | 566 | 4.0 | 426 | 3.1 | 5.1 |
| Diseases of the musculoskeletal system and connective tissue | 401 | 2.9 | 234 | 1.7 | 5.9 |
| Mental and behavioural disorders | 399 | 2.8 | 310 | 2.2 | 3.0 |
| Neoplasms | 168 | 1.195 | 80 | 0.6 | 1.4 |

Note: Subgroups within ICD-10 chapters are indented.

*Age standardized for age groups 0-24 using the 2013 European standard population per 1000 person-years.

**For obstetrics and medical abortion, the percent of children and the age-standardised rate are calculated for the female members of the cohort.

For peer review only

*Figure 1. The development and size of the two study cohorts*

For peer review only



For peer review only - http://bmjopen.bmj.com/site/about/guidelines.xhtml

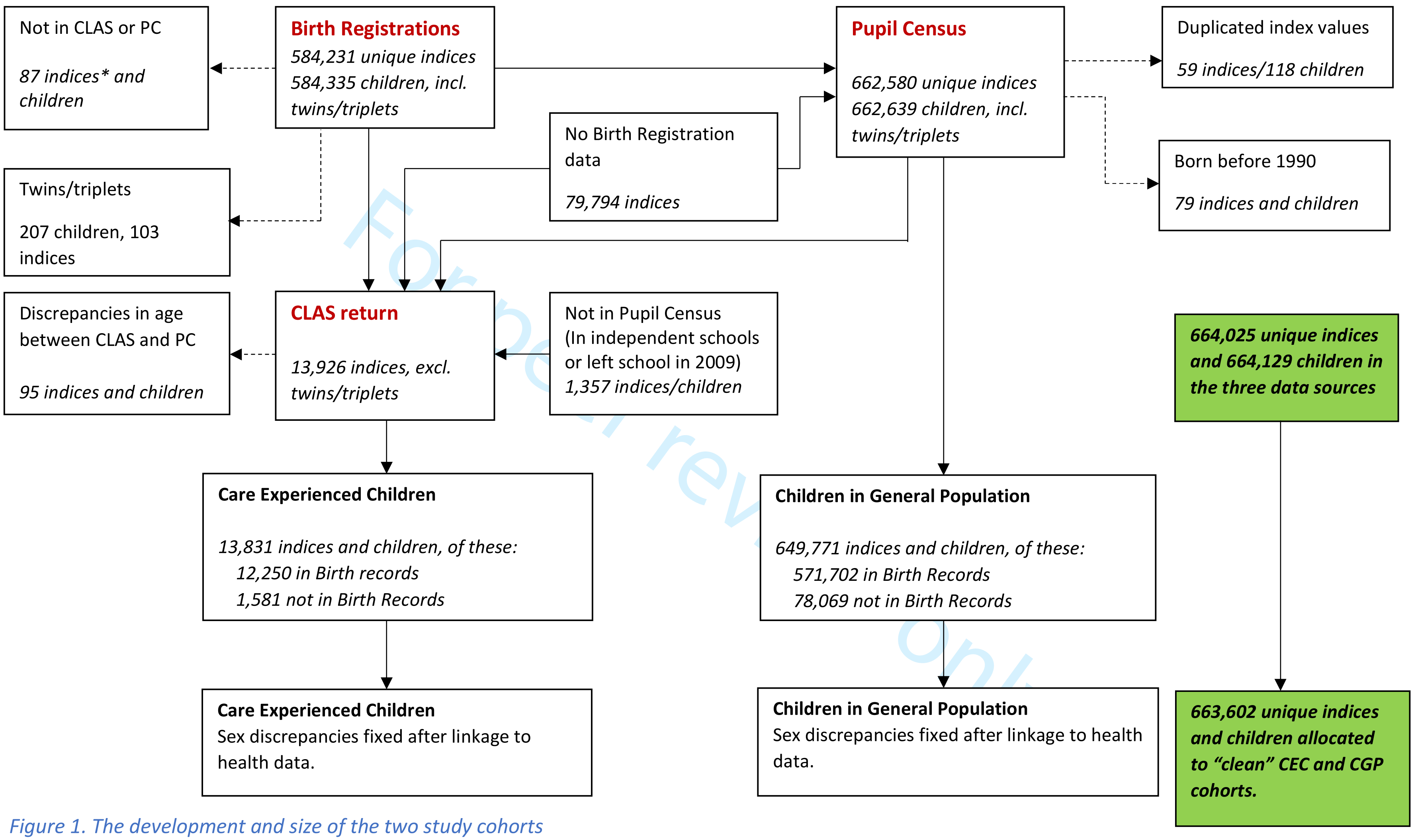


*Figure 1. The development and size of the two study cohorts*

*Each index refers to a unique child. When there are more index values than children in the data (in Birth Registrations and Pupil Census) this means that more than one child had the same linkage identifiers (date of birth, postcode, sex). This is the case for twins/triplets and other linkage errors, these duplicated index values were removed from the data as it was not possible to determine which health records belonged to which child.

Note: Dashed arrows indicate children who were removed from the cohorts during data cleaning.

## Supplement

*Table S.1: Health outcomes of interest*

| Data set | Prior health | Outcome measures | Total events 2009-2016 *N* Valid | % Valid* |
|---|---|---|---|---|
| Death Records | NA | Number of deaths | | |
| | | Cause of death | 824 | 100 |
| PIS - Prescribing Information System | No | Number of prescriptions | | |
| | | BNF chapter/section | 17,533,253 | 99.3 |
| SMR00 - Outpatient Attendance | Yes | Number of admissions | | |
| | | Speciality | 2,487,792 | 100 |
| SMR01 - General/ Acute Inpatient and Day Case | Yes | Number of admissions | | |
| | | Condition diagnosed | 412,616 | 100 |
| SMR02 - Maternity Inpatient and Day Case | Yes | Number of admissions | | |
| | | Condition diagnosed | 44,447 | 100 |
| | | *Admission reason* | | 100 |
| SMR04 - Mental Health Inpatient and Day Case | Yes | Number of admissions | | |
| | | Condition diagnosed | 8,774 | 99.9 |
| | | *Admission reason* | | 83.8 |
| A&E - Accidents and Emergencies | Yes | Number of attendances per year | 1,523,815 | NA |

*Percent with valid code for BNF chapter/section, diagnosed condition or admission reason from total recorded events. Not applicable for A&E attendances.



For peer review only

*Table S.2: Urban-Rural Classification and Health Board of residence of CEC and CGP cohorts*

| | **All children with birth records** *Area of residence at birth* | | | | **All children with birth records** *Area of residence in 2009* | | | | **All children** *Area of residence in 2009* | | | |
|---|---|---|---|---|---|---|---|---|---|---|---|---|
| | **CGP** | | **CEC** | | **CGP** | | **CEC** | | **CGP** | | **CEC** | |
| | **N** | **%** | **N** | **%** | **N** | **%** | **N** | **%** | **N** | **%** | **N** | **%** |
| **Number (%) of children** | **571,702** | **88.0** | **12,250** | **88.6** | **571,702** | **88.0** | **12,250** | **88.6** | **649,771** | **100.0** | **13,831** | **100.0** |
| **Urban-Rural classification** | | | | | | | | | | | | |
| Large urban areas | 220,713 | 38.6 | 6,164 | 50.3 | 199,694 | 34.9 | 4,853 | 39.6 | 226,405 | 34.8 | 5,349 | 38.7 |
| Other urban areas | 172,673 | 30.2 | 3,580 | 29.2 | 188,845 | 33.0 | 3,509 | 28.6 | 209,340 | 32.2 | 3,893 | 28.1 |
| Accessible small towns | 58,649 | 10.3 | 1,012 | 8.3 | 52,500 | 9.2 | 861 | 7.0 | 59,155 | 9.1 | 944 | 6.8 |
| Remote small towns | 15,772 | 2.8 | 363 | 3.0 | 22,037 | 3.9 | 460 | 3.8 | 25,424 | 3.9 | 505 | 3.7 |
| Accessible rural | 74,024 | 12.9 | 823 | 6.7 | 72,390 | 12.7 | 927 | 7.6 | 83,801 | 12.9 | 1,068 | 7.7 |
| Remote rural | 29,013 | 5.1 | 291 | 2.4 | 36,011 | 6.3 | 457 | 3.7 | 45,008 | 6.9 | 562 | 4.1 |
| *Missing* | *858* | *0.2* | *17* | *0.1* | *225* | 0.0 | *1,183* | 9.7 | *638* | 0.1 | *1,510* | 10.9 |
| **Health Boards*** | | | | | | | | | | | | |
| Ayrshire & Clyde | 46,869 | 8.2 | 1,356 | 11.1 | - | - | - | - | - | - | - | - |
| Ayrshire & Arran | 41,008 | 7.2 | 952 | 7.8 | 43,278 | 7.6 | 958 | 7.8 | 47,621 | 7.3 | 1,072 | 7.8 |
| Borders | 10,681 | 1.9 | 131 | 1.1 | 12,031 | 2.1 | 144 | 1.2 | 14,663 | 2.3 | 165 | 1.2 |
| Dumfries & Galloway | 15,581 | 2.7 | 261 | 2.1 | 16,037 | 2.8 | 263 | 2.1 | 19,064 | 2.9 | 304 | 2.2 |
| Fife | 39,279 | 6.9 | 640 | 5.2 | 41,013 | 7.2 | 712 | 5.8 | 45,907 | 7.1 | 796 | 5.8 |
| Forth Valley | 31,866 | 5.6 | 716 | 5.8 | 33,848 | 5.9 | 676 | 5.5 | 37,965 | 5.8 | 753 | 5.4 |
| Grampian | 54,813 | 9.6 | 1,157 | 9.4 | 55,016 | 9.6 | 1,013 | 8.3 | 65,622 | 10.1 | 1,128 | 8.2 |
| Greater Glasgow (&Clyde) | 103,106 | 18.0 | 2,889 | 23.6 | 132,673 | 23.2 | 3,242 | 26.5 | 148,650 | 22.9 | 3,577 | 25.9 |
| Highland | 24,089 | 4.2 | 390 | 3.2 | 34,323 | 6.0 | 552 | 4.5 | 41,643 | 6.4 | 636 | 4.6 |
| Lanarkshire | 70,439 | 12.3 | 935 | 7.6 | 73,274 | 12.8 | 1,041 | 8.5 | 89,242 | 13.7 | 1,142 | 8.3 |
| Lothian | 83,929 | 14.7 | 1,833 | 15.0 | 79,506 | 13.9 | 1,595 | 13.0 | 90,757 | 14.0 | 1,755 | 12.7 |
| Orkney | 2,145 | 0.4 | 21 | 0.2 | 2,206 | 0.4 | 23 | 0.2 | 2,709 | 0.4 | 29 | 0.2 |
| Shetland | 2,789 | 0.5 | 27 | 0.2 | 2,754 | 0.5 | 24 | 0.2 | 3,283 | 0.5 | 27 | 0.2 |
| Tayside | 41,514 | 7.3 | 893 | 7.3 | 42,602 | 7.5 | 787 | 6.4 | 48,465 | 7.5 | 891 | 6.4 |
| Western Isles | 2,736 | 0.5 | 32 | 0.3 | 2,916 | 0.5 | 37 | 0.3 | 3,557 | 0.5 | 46 | 0.3 |
| *Missing* | *858* | *0.2* | *17* | *0.1* | *225* | *0.0* | *1,183* | *9.7* | *638* | 0.1 | *1,510* | 10.9 |

*Health Board at birth based on 1995 Health Boards. Health Board in 2009 based on 2006 Health Boards. In 2006, NHS Argyll and Clyde responsibilities were shared between NHS Highland and NHS Greater Glasgow.



For peer review only - http://bmjopen.bmj.com/site/about/guidelines.xhtml

*Table S.3: Residential stability of datazone between birth and 2009*

| | CGP with birth records | | CEC with birth records | |
|---|---|---|---|---|
| | N | % | N | % |
| | 571,702 | | 12,250 | |
| Same data zone at birth and in 2009 | 215,308 | 37.7 | 1,523 | 12.4 |
| Moved data zone between birth and 2009 | 355,324 | 62.2 | 9,527 | 77.8 |
| *Missing** | *1,070* | *0.2* | *1,200* | *9.8* |

*Of data zones missing in the CGP cohort: 858 are missing at birth, 225 are missing in 2009 and 13 are missing in both periods. Of data zones missing in the CEC cohort: 17 are missing at birth and 1183 are missing in 2009.

*Table S.4: Comparison of CEC cohort to national rates of children looked after by sex and age*

| | CEC cohort: Children on 2009/10 CLAS return and 2009 PC | | National rates of Children Looked After* | | | | CEC cohort as % from national rates of Children Looked After |
|---|---|---|---|---|---|---|---|
| | N | % | A) Looked after at 31 March 2009 | B) Starting to be looked after 01/08/2009 - 31/07/2010 | A + B | % | |
| Female | 6,274 | 45.4 | 6,912 | 2,288 | 9,200 | 45.2 | 68.2 |
| Male | 7,557 | 54.6 | 8,375 | 2,571 | 10,946 | 54.8 | 69.0 |
| Age (at 31st July 2009) | | | | | | | |
| 0-4 | 336 | 2.4 | 3,157 | 1,796 | 4,953 | 20.7 | 6.8 |
| 5 to 11 | 6,670 | 48.2 | 5,510 | 1,463 | 6,973 | 36 | 95.7 |
| 12 to 15 | 5,845 | 42.3 | 5,017 | 1,555 | 6,572 | 32.8 | 88.9 |
| 16 & 17 | 956 | 6.9 | 1,457 | 45 | 1,502 | 9.5 | 63.6 |
| 18+ | 24 | 0.2 | 146 | 0 | 146 | 1 | 16.4 |
| Total | 13,831 | 100 | 15,287 | 4,859 | 20,146 | 100 | 68.7 |

*Source: https://www.gov.scot/publications/statistics-publication-notice-health-care-series-children-looked-statistics-2009-10/pages/1/



For peer review only - http://bmjopen.bmj.com/site/about/guidelines.xhtml

*Table S.5: Comparison of all children in study (CEC and CGP) to Scotland's 2011 census population, age 0-19 years*

| | **All children in study** | | **Scotland's census 2011**** | | |
|---|---|---|---|---|---|
| | **N** | **%** | **N** | **%** | |
| **All children in study (CEC and CGP), at 31 July 2009*** | **663,602** | | | | |
| **All children in Scotland at 2011 census** | | | **1,184,879** | | |
| | | | | | *% of census population* |
| **Female** | 325,712 | 49.1 | 579,949 | 48.9 | *56.2* |
| **Male** | 337,890 | 50.9 | 604,930 | 51.1 | *55.9* |
| | | | | | |
| **Age** | | | | | |
| 0-4 | 20,957 | 3.2 | 292,821 | 24.7 | *7.2* |
| 5 to 11 | 364,271 | 54.9 | 381,453 | 32.2 | *95.5* |
| 12 to 15 | 226,091 | 34.1 | 242,057 | 20.4 | *93.4* |
| 16 & 17 | 51,964 | 7.8 | 126,266 | 10.7 | *41.2* |
| 18 & 19 | 319 | 0.0 | 142,282 | 12.0 | *0.2* |

*Includes only those children in publicly-funded schools.
**Source: Scotland's Census 2011. Table DC1117SC – Age by sex, All people



For peer review only

*Table S.6: Coding schemas for SMR00 and SMR01*

| Label used in tables | Codes |
|---|---|
| **Clinic specialty SMR00** | **Specialty/Discipline Codes included*** |
| Psychiatry | G1, G1A, G2, G21, G22, G3, G4 |
| Dentistry | D6, D61-D63, D8, DC, DE, DD |
| Obstetrics | E11, F3, F31, F32 |
| | |
| **Main condition SMR01** | **ICD-10 codes included** |
| Diseases of the digestive system | K00-K93 |
| Injury, poisoning and certain other consequences of external causes | S00-T98 |
| Other injury | S10-S59, S70-T35, T51-T98 |
| Head injury | S00-S09 |
| Drug poisoning | T36-T50 |
| Hand injury | S60-S69 |
| | |
| Symptoms, signs and abnormal clinical and laboratory findings | R00-R99 |
| Diseases of the respiratory system | J00-J99 |
| Asthma | =J45-J46 |
| | |
| Diseases of the genitourinary system | N00-N99 |
| | |
| Diseases of the musculoskeletal system and connective tissue | M00-M99 |
| Neoplasms | C00-D48 |
| Examination, observation, etc | Z00-Z99 |
| Obstetric | O00-O99 |
| | |
| Endocrine, nutritional and metabolic diseases | E00-E90 |
| Mental and behavioural disorders | F00-F99 |

*Clinic specialty codes are described in the SMR00 crib sheet https://www.ndc.scot.nhs.uk/Data-Dictionary/SMR-Crib-Sheets/SMR00_CRIB_07012021.pdf



For peer review only

For peer review only - http://bmjopen.bmj.com/site/about/guidelines.xhtml

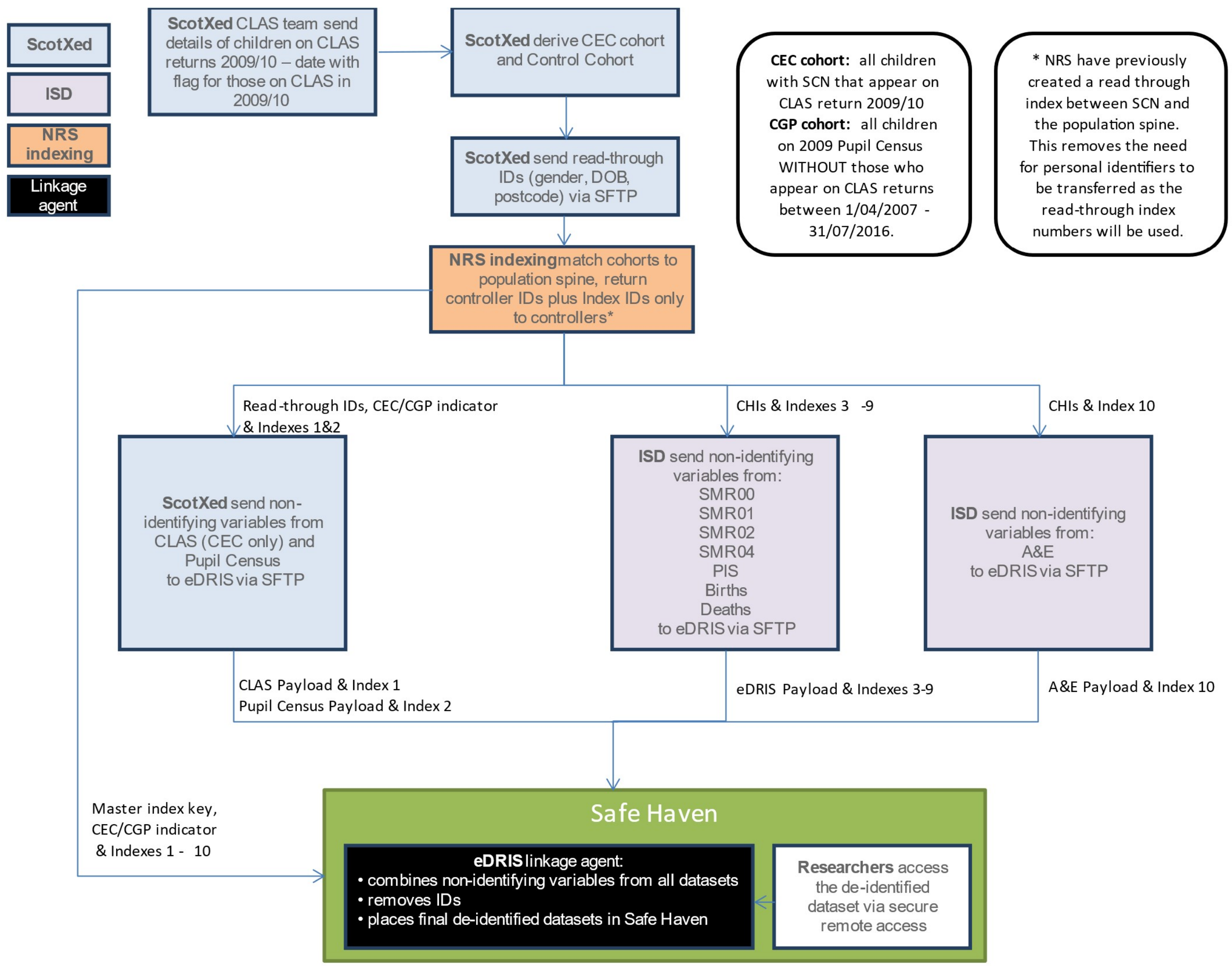


*Figure S.1. Linkage diagram for the CHiCS project*

CEC – care experienced children; CGP – children in the general population; SFTP – secure file transfer protocol; ScotXed – Scottish Exchange of Data; NRS – National Records of Scotland; ISD – Information Services Division (part of NHS); eDRIS – electronic Data Research and Innovation Service; SCN – Scottish Candidate Number



For peer review only - http://bmjopen.bmj.com/site/about/guidelines.xhtml
